# Modeling SAGE tag formation and its effects on data interpretation within a Bayesian framework

**DOI:** 10.1186/1471-2105-8-403

**Published:** 2007-10-18

**Authors:** Michael A Gilchrist, Hong Qin, Russell Zaretzki

**Affiliations:** 1 Department of Ecology and Evolutionary Biology, University of Tennessee, Knoxville, TN 37996, USA; 2 Department of Statistics, University of Tennessee, Knoxville, TN 37996, USA; 3 Dept of Agricultural and Environmental Sciences, Tuskegee University, Tuskegee, AL 36088, USA

## Abstract

**Background:**

Serial Analysis of Gene Expression (SAGE) is a high-throughput method for inferring mRNA expression levels from the experimentally generated sequence based tags. Standard analyses of SAGE data, however, ignore the fact that the probability of generating an observable tag varies across genes and between experiments. As a consequence, these analyses result in biased estimators and posterior probability intervals for gene expression levels in the transcriptome.

**Results:**

Using the yeast *Saccharomyces cerevisiae *as an example, we introduce a new Bayesian method of data analysis which is based on a model of SAGE tag formation. Our approach incorporates the variation in the probability of tag formation into the interpretation of SAGE data and allows us to derive exact joint and approximate marginal posterior distributions for the mRNA frequency of genes detectable using SAGE. Our analysis of these distributions indicates that the frequency of a gene in the tag pool is influenced by its mRNA frequency, the cleavage efficiency of the anchoring enzyme (AE), and the number of informative and uninformative AE cleavage sites within its mRNA.

**Conclusion:**

With a mechanistic, model based approach for SAGE data analysis, we find that inter-genic variation in SAGE tag formation is large. However, this variation can be estimated and, importantly, accounted for using the methods we develop here. As a result, SAGE based estimates of mRNA frequencies can be adjusted to remove the bias introduced by the SAGE tag formation process.

## Background

The Serial Analysis of Gene Expression (SAGE) is a high-throughput method to quantify the distribution of mRNA transcripts in a biological sample by sequencing a large set of tags [1]. As part of the process of generating tags, the SAGE method uses a restriction enzyme, termed the Anchoring Enzyme (AE), to cleave the double-stranded cDNA derived from mRNA transcripts. Cleavage by the AE generates sequence tags that are 3' adjacent to the cleavage site. Depending on the specific technique used, tags generally range from 10 to 20 base pairs in length. Tags are then concatenated into long fragments. This allows for the identification of multiple tags in a single sequencing reaction. In general, increasing tag length increases the probability that a tag can be unambiguously attributed to the transcript of a single gene. Such tags are considered to be 'informative' while tags which cannot be unambiguously attributed to a single gene (ambiguous tags) are 'uninformative' using current techniques.

As with all empirical techniques, SAGE has a number of technical disadvantages and advantages. Tag sampling can be thought of as a multinomial sampling process [2] where the distribution of a focal tag follows a binomial distribution [3,4] or is approximated with a Poisson distribution [5-8]. Because SAGE is a sampling based technique and only a limited number of tags are sequenced, sampling error is a major source of noise in SAGE data. Consequently, SAGE provides little information on genes with low expression levels [2,9]. The uncertainty caused by limited sample sizes has been addressed by several methods. For example, [10] use a hierarchical Poisson mixture approach to deal with the uncertainty associated with genes with low expression levels, while [11] utilizes a mixture model to adjust for differences between genes with high and low expression levels. Other approaches have been developed to improve detection of differences between samples [6,12,13].

Another factor regarding the quality of SAGE data is ascertainment errors. For example, the use of PCR to amplify mRNA samples can introduce copying errors into tag sequences. Tags are identified via DNA sequencing, which is an imperfect processes. Depending on the tag's length, approximately 7–14% of all tags contain sequencing errors [9]. Consequently, a number of sophisticated techniques have been developed to correct for such errors [14-16].

In terms of advantages, in contrast to other methods for inferring mRNA expression levels, SAGE is useful for identifying actual genes, as opposed to pseudo-genes. Further, because SAGE measurements do not rely on florescence measurements, they do not suffer from saturation effects. As a result, SAGE data are considered to be more accurate than hybridization-based measurements for genes with high expression levels [17]. Furthermore, as the cost of sequencing decreases and the accuracy of its measurements increases, SAGE may become increasingly advantageous [18].

Independent of these strengths and weaknesses, one common aspect of current SAGE data analyses which has not been questioned is the implicit assumption that observed tag frequencies are suitable estimates of mRNA frequencies. However, as our results will demonstrate below, differences exist among genes in the probabilities of tag formation from their mRNA transcripts. Such differences must be taken into account in order to accurately estimate expression levels.

The probability of tag formation from an individual mRNA transcript is determined by the number of unambiguous tags formed from the AE sites in its transcript and the cleavage efficiency of AE in a given experiment. Because the number of AE sites can vary between genes and not all tags are unambiguous (i.e. informative), the probability of detecting a transcript can vary greatly from gene to gene. A clear case of such differences in tag formation probabilities is illustrated by considering genes without any AE sites. Because tag formation is dependent on AE cleavage, mRNA lacking such sites have zero probability of forming tags and, consequently, cannot be detected by SAGE. Our work extends this idea by recognizing that even amongst the set of genes with AE cleavage sites, not all genes are equally likely to form unambiguous, informative SAGE tags. We find that the probability of forming an informative tag is a complex function of the number of AE sites, the cutting efficiency of the AE, and the uniqueness of the tags produced. We also point out that a high AE cutting efficiency may not always be desirable.

Because there is inter-genic variation in tag formation probabilities, the proportion of tags in the population sampled experimentally (or tag pool, for short) is not equivalent to the proportion of a particular mRNA transcript in the cell (or mRNA pool, which is the actual pool of interest). This difference has, until now, been ignored by the current methods for SAGE data analysis. (Hereafter, we refer to such methods as "standard methods"). In contrast, the proposed method recognizes the difference between the observed tag frequencies and the actual frequency of transcripts. More specifically, we formally link tag and mRNA pools using a mechanistic model of how tags are formed from mRNA transcripts which naturally incorporates the tag formation probability of each gene. Intrinsically, genes with lower than average tag formation probabilities are underestimated while the converse holds for genes with higher than average tag formation probabilities. Given that tag formation probabilities are positively correlated with gene length, which in turn is negatively correlated with expression level, we find that the bias introduced by during tag formation leads to systematic under- and over-estimation of lowly and highly expressed genes, respectively. Fortunately, by taking the differences in tagging probability among genes into account, we can remove such biases and, thereby, increase the quality of inferences made from SAGE data.

## Results

The purpose of our study is to incorporate differences in the probability of tag formation into the analysis of SAGE data. We developed a formal framework for incorporating these differences, derive exact and approximate solutions, and then illustrate the framework's utility by applying it to a published *Saccharomyces cerevisiae *SAGE data set [9]. The output from the analysis such as summary statistics, modal values, posterior percentiles, and 95%PI can be found in Additional Files 1, 2, 3, 4, 5, 6.

### Model formulation

#### Tag sampling & generation

**Figure 1:**
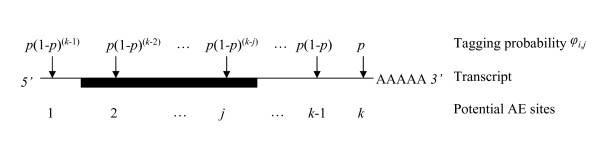
Diagram of hypothetical mRNA transcript with its potential AE cut sites (indicated by arrows) and their tag formation probability *φ*_*j*_. AE sites are assumed to be cleaved independently of one another with cleaving efficiency *p*. From an individual mRNA, the tag formed is from the 3' most AE site that is actually cleaved. The probability of forming a tag at the *j*^th ^site is, therefore, *p*(1 - *p*)^(*k - j*)^.

The generation of a SAGE data set is frequently viewed as a multinomial sampling process in which tags are sampled from a tag pool. The generation of the tag pool from the mRNA pool relies on the AE cleavage of the cDNA copy of mRNA transcripts. For simplicity, we assume that the cleavage probability is constant for all sites and that all cleavage events are independent of one another. We use *p *to denote the average cleavage efficiency of the AE and ^*k*^*i *to denote the number of cleavage sites within an mRNA transcript of gene *i *(Figure 1). Later, when applying the model and in the Appendix A, we explain how it is possible to estimate *p *from the observed intra-genic distributions of tags.

#### Formation of the tag pool from the mRNA pool

To derive the relationship between the tag and mRNA pools, we begin by defining the mRNA pool and the tag pool explicitly. The tag pool represents the set of informative tags which can be unambiguously assigned to specific genes. The mRNA pool is the subset of the transcriptome which, through AE cleavage, can generate at least one informative tag. For simplicity, we assume that each gene generates only one kind of transcript (i.e. we ignore any alternative splicing). We represent the mRNA pool that we are trying to estimate as m→
 MathType@MTEF@5@5@+=feaafiart1ev1aaatCvAUfKttLearuWrP9MDH5MBPbIqV92AaeXatLxBI9gBaebbnrfifHhDYfgasaacH8akY=wiFfYdH8Gipec8Eeeu0xXdbba9frFj0=OqFfea0dXdd9vqai=hGuQ8kuc9pgc9s8qqaq=dirpe0xb9q8qiLsFr0=vr0=vr0dc8meaabaqaciaacaGaaeqabaqabeGadaaakeaacuWGTbqBgaWcaaaa@2E21@ = {*m*_1_, *m*_2_, ..., *m*_*n*_}, where *m*_*i *_represents the proportion of the *i*^th ^gene out of *n *genes detectable by the SAGE method. By definition, ∑i=1nmi=1
 MathType@MTEF@5@5@+=feaafiart1ev1aaatCvAUfKttLearuWrP9MDH5MBPbIqV92AaeXatLxBI9gBaebbnrfifHhDYfgasaacH8akY=wiFfYdH8Gipec8Eeeu0xXdbba9frFj0=OqFfea0dXdd9vqai=hGuQ8kuc9pgc9s8qqaq=dirpe0xb9q8qiLsFr0=vr0=vr0dc8meaabaqaciaacaGaaeqabaqabeGadaaakeaadaaeWaqaaiabd2gaTnaaBaaaleaacqWGPbqAaeqaaaqaaiabdMgaPjabg2da9iabigdaXaqaaiabd6gaUbqdcqGHris5aOGaeyypa0JaeGymaedaaa@3843@ (Note that symbols used in this study are listed in Table 1). The tag pool in turn is defined as θ→
 MathType@MTEF@5@5@+=feaafiart1ev1aaatCvAUfKttLearuWrP9MDH5MBPbIqV92AaeXatLxBI9gBaebbnrfifHhDYfgasaacH8akY=wiFfYdH8Gipec8Eeeu0xXdbba9frFj0=OqFfea0dXdd9vqai=hGuQ8kuc9pgc9s8qqaq=dirpe0xb9q8qiLsFr0=vr0=vr0dc8meaabaqaciaacaGaaeqabaqabeGadaaakeaaiiGacuWF4oqCgaWcaaaa@2E7B@ = {*θ*_1_, *θ*_2_, ..., *θ*_*n*_}, where *θ*_*i *_is the expected proportion of tags from gene *i *in the tag pool. The tag proportion of gene *i*, *θ*_*i*_, is the sum of the individual proportions of all informative tags generated from the transcript of the *i*^th ^gene. As with the mRNA pool, by definition, ∑i=1nθi=1
 MathType@MTEF@5@5@+=feaafiart1ev1aaatCvAUfKttLearuWrP9MDH5MBPbIqV92AaeXatLxBI9gBaebbnrfifHhDYfgasaacH8akY=wiFfYdH8Gipec8Eeeu0xXdbba9frFj0=OqFfea0dXdd9vqai=hGuQ8kuc9pgc9s8qqaq=dirpe0xb9q8qiLsFr0=vr0=vr0dc8meaabaqaciaacaGaaeqabaqabeGadaaakeaadaaeWaqaaGGaciab=H7aXnaaBaaaleaacqWGPbqAaeqaaaqaaiabdMgaPjabg2da9iabigdaXaqaaiabd6gaUbqdcqGHris5aOGaeyypa0JaeGymaedaaa@389D@. Our next step is to understand how the tag and mRNA pool frequencies are linked.

**Table 1:** Symbol Definitions

Symbol	Definitions
*n*	Total number of genes with potential AE sites in a transcriptome.
*k*, *k*_*i*_	Total number of AE cleavage sites within the transcripts of a gene (or gene *i*).
k′i MathType@MTEF@5@5@+=feaafiart1ev1aaatCvAUfKttLearuWrP9MDH5MBPbIqV92AaeXatLxBI9gBaebbnrfifHhDYfgasaacH8akY=wiFfYdH8Gipec8Eeeu0xXdbba9frFj0=OqFfea0dXdd9vqai=hGuQ8kuc9pgc9s8qqaq=dirpe0xb9q8qiLsFr0=vr0=vr0dc8meaabaqaciaacaGaaeqabaqabeGadaaakeaacuWGRbWAgaqbamaaBaaaleaacqWGPbqAaeqaaaaa@2F9E@	Total number of AE cleavage sites within the coding region of gene *i*.
*p*	Global cleavage efficiency of the AE.
*m*_*i*_	The frequency of mRNA for the *i*^th ^gene in the transcriptome. ∑i=1nmi=1 MathType@MTEF@5@5@+=feaafiart1ev1aaatCvAUfKttLearuWrP9MDH5MBPbIqV92AaeXatLxBI9gBaebbnrfifHhDYfgasaacH8akY=wiFfYdH8Gipec8Eeeu0xXdbba9frFj0=OqFfea0dXdd9vqai=hGuQ8kuc9pgc9s8qqaq=dirpe0xb9q8qiLsFr0=vr0=vr0dc8meaabaqaciaacaGaaeqabaqabeGadaaakeaadaaeWaqaaiabd2gaTnaaBaaaleaacqWGPbqAaeqaaaqaaiabdMgaPjabg2da9iabigdaXaqaaiabdMeajbqdcqGHris5aOGaeyypa0JaeGymaedaaa@37F9@.
m→ MathType@MTEF@5@5@+=feaafiart1ev1aaatCvAUfKttLearuWrP9MDH5MBPbIqV92AaeXatLxBI9gBaebbnrfifHhDYfgasaacH8akY=wiFfYdH8Gipec8Eeeu0xXdbba9frFj0=OqFfea0dXdd9vqai=hGuQ8kuc9pgc9s8qqaq=dirpe0xb9q8qiLsFr0=vr0=vr0dc8meaabaqaciaacaGaaeqabaqabeGadaaakeaacuWGTbqBgaWcaaaa@2E21@	{*m*_1_, *m*_2_, ..., *m*_*n*_}
m^i MathType@MTEF@5@5@+=feaafiart1ev1aaatCvAUfKttLearuWrP9MDH5MBPbIqV92AaeXatLxBI9gBaebbnrfifHhDYfgasaacH8akY=wiFfYdH8Gipec8Eeeu0xXdbba9frFj0=OqFfea0dXdd9vqai=hGuQ8kuc9pgc9s8qqaq=dirpe0xb9q8qiLsFr0=vr0=vr0dc8meaabaqaciaacaGaaeqabaqabeGadaaakeaacuWGTbqBgaqcamaaBaaaleaacqWGPbqAaeqaaaaa@2FA6@	mRNA frequency of gene *i *the joint posterior mode.
m˜i MathType@MTEF@5@5@+=feaafiart1ev1aaatCvAUfKttLearuWrP9MDH5MBPbIqV92AaeXatLxBI9gBaebbnrfifHhDYfgasaacH8akY=wiFfYdH8Gipec8Eeeu0xXdbba9frFj0=OqFfea0dXdd9vqai=hGuQ8kuc9pgc9s8qqaq=dirpe0xb9q8qiLsFr0=vr0=vr0dc8meaabaqaciaacaGaaeqabaqabeGadaaakeaacuWGTbqBgaacamaaBaaaleaacqWGPbqAaeqaaaaa@2FA5@	mRNA frequency of gene *i *at the marginal posterior mode.
*θ*_*i*_	The frequency of the observed tags for the *i*^th ^gene in the total tag pool, ∑i=1nθi=1 MathType@MTEF@5@5@+=feaafiart1ev1aaatCvAUfKttLearuWrP9MDH5MBPbIqV92AaeXatLxBI9gBaebbnrfifHhDYfgasaacH8akY=wiFfYdH8Gipec8Eeeu0xXdbba9frFj0=OqFfea0dXdd9vqai=hGuQ8kuc9pgc9s8qqaq=dirpe0xb9q8qiLsFr0=vr0=vr0dc8meaabaqaciaacaGaaeqabaqabeGadaaakeaadaaeWaqaaGGaciab=H7aXnaaBaaaleaacqWGPbqAaeqaaaqaaiabdMgaPjabg2da9iabigdaXaqaaiabd6gaUbqdcqGHris5aOGaeyypa0JaeGymaedaaa@389D@.
θ→ MathType@MTEF@5@5@+=feaafiart1ev1aaatCvAUfKttLearuWrP9MDH5MBPbIqV92AaeXatLxBI9gBaebbnrfifHhDYfgasaacH8akY=wiFfYdH8Gipec8Eeeu0xXdbba9frFj0=OqFfea0dXdd9vqai=hGuQ8kuc9pgc9s8qqaq=dirpe0xb9q8qiLsFr0=vr0=vr0dc8meaabaqaciaacaGaaeqabaqabeGadaaakeaaiiGacuWF4oqCgaWcaaaa@2E7B@	{*θ*_1_, *θ*_2_, ..., *θ*_*n*_}
θ^i MathType@MTEF@5@5@+=feaafiart1ev1aaatCvAUfKttLearuWrP9MDH5MBPbIqV92AaeXatLxBI9gBaebbnrfifHhDYfgasaacH8akY=wiFfYdH8Gipec8Eeeu0xXdbba9frFj0=OqFfea0dXdd9vqai=hGuQ8kuc9pgc9s8qqaq=dirpe0xb9q8qiLsFr0=vr0=vr0dc8meaabaqaciaacaGaaeqabaqabeGadaaakeaaiiGacuWF4oqCgaqcamaaBaaaleaacqWGPbqAaeqaaaaa@3000@	Tag frequency of gene *i *the joint posterior mode.
*φ*_*i*_	Tag formation probability of gene *i*. Note this varies by experiment.
Φ¯ MathType@MTEF@5@5@+=feaafiart1ev1aaatCvAUfKttLearuWrP9MDH5MBPbIqV92AaeXatLxBI9gBaebbnrfifHhDYfgasaacH8akY=wiFfYdH8Gipec8Eeeu0xXdbba9frFj0=OqFfea0dXdd9vqai=hGuQ8kuc9pgc9s8qqaq=dirpe0xb9q8qiLsFr0=vr0=vr0dc8meaabaqaciaacaGaaeqabaqabeGadaaakeaacuqHMoGrgaqeaaaa@2E3E@	Mean tag formation progbability which is the sum of *φ*_*i*_*m*_*i *_across all genes.
*T*_*i*_, *T*_*i, k*_	Number of observed tags (at the *j*^th ^AE site) within the *i*^th ^gene's transcript.
T→ MathType@MTEF@5@5@+=feaafiart1ev1aaatCvAUfKttLearuWrP9MDH5MBPbIqV92AaeXatLxBI9gBaebbnrfifHhDYfgasaacH8akY=wiFfYdH8Gipec8Eeeu0xXdbba9frFj0=OqFfea0dXdd9vqai=hGuQ8kuc9pgc9s8qqaq=dirpe0xb9q8qiLsFr0=vr0=vr0dc8meaabaqaciaacaGaaeqabaqabeGadaaakeaacuWGubavgaWcaaaa@2DEF@	{*T*_1_, *T*_2_, ..., *T*_*n*_}
*T*_0_	Total number of observed informative tags, which is ∑_*i*_∑_*j*_*T*_*i*, *j*_
*α*_*i*_	Parameter for the prior of *m*_*i*_.
α→ MathType@MTEF@5@5@+=feaafiart1ev1aaatCvAUfKttLearuWrP9MDH5MBPbIqV92AaeXatLxBI9gBaebbnrfifHhDYfgasaacH8akY=wiFfYdH8Gipec8Eeeu0xXdbba9frFj0=OqFfea0dXdd9vqai=hGuQ8kuc9pgc9s8qqaq=dirpe0xb9q8qiLsFr0=vr0=vr0dc8meaabaqaciaacaGaaeqabaqabeGadaaakeaaiiGacuWFXoqygaWcaaaa@2E64@	{*α*_1_, *α*_2_, ..., *α*_*n*_}
*α*_0_	Sum of all prior parameters, i.e. ∑_*i *_*α*_*i*_.
*β*_*i*_	Sum of prior parameters for genes other than *i*, i.e. *α*_0 _- *α*_*i*_.

In order for a tag to form at a particular AE site, say the *j*^th ^site, the AE must cut the cDNA derived from an mRNA transcript at the *j*^th ^site but not at any of the other sites between 3' and the *j*^th ^site. If there are *k*_*i *_potential AE sites in the *i*^th ^gene, then it follows that the probability of forming a tag at the *j*^th ^site is

φi,j=p(1−p)ki−j.
 MathType@MTEF@5@5@+=feaafiart1ev1aaatCvAUfKttLearuWrP9MDH5MBPbIqV92AaeXatLxBI9gBaebbnrfifHhDYfgasaacH8akY=wiFfYdH8Gipec8Eeeu0xXdbba9frFj0=OqFfea0dXdd9vqai=hGuQ8kuc9pgc9s8qqaq=dirpe0xb9q8qiLsFr0=vr0=vr0dc8meaabaqaciaacaGaaeqabaqabeGadaaakeaaiiGacqWFgpGzdaWgaaWcbaGaemyAaKMaeiilaWIaemOAaOgabeaakiabg2da9iabdchaWjabcIcaOiabigdaXiabgkHiTiabdchaWjabcMcaPmaaCaaaleqabaGaem4AaS2aaSbaaWqaaiabdMgaPbqabaWccqGHsislcqWGQbGAaaGccqGGUaGlaaa@3FF8@

The first term *p *represents the probability that AE cleaves a transcript at the *j*^th ^site. The second term (1−p)(ki−j)
 MathType@MTEF@5@5@+=feaafiart1ev1aaatCvAUfKttLearuWrP9MDH5MBPbIqV92AaeXatLxBI9gBaebbnrfifHhDYfgasaacH8akY=wiFfYdH8Gipec8Eeeu0xXdbba9frFj0=OqFfea0dXdd9vqai=hGuQ8kuc9pgc9s8qqaq=dirpe0xb9q8qiLsFr0=vr0=vr0dc8meaabaqaciaacaGaaeqabaqabeGadaaakeaacqGGOaakcqaIXaqmcqGHsislcqWGWbaCcqGGPaqkdaahaaWcbeqaaiabcIcaOiabdUgaRnaaBaaameaacqWGPbqAaeqaaSGaeyOeI0IaemOAaOMaeiykaKcaaaaa@38BF@ represents the probability of the AE *not *cleaving a transcript between the (*k*_*i *_- *j*) sites 3' to the *j*^th ^site. The next step is to calculate the total probability that mRNA transcripts for a given gene will be converted into informative SAGE tags.

Because we assume that each site is cleaved independently, the total probability of tags being formed from mRNA transcripts for a given gene is simply the sum of the cleavage probabilities for all possible informative tags. That is

φi=∑jφi,j,
 MathType@MTEF@5@5@+=feaafiart1ev1aaatCvAUfKttLearuWrP9MDH5MBPbIqV92AaeXatLxBI9gBaebbnrfifHhDYfgasaacH8akY=wiFfYdH8Gipec8Eeeu0xXdbba9frFj0=OqFfea0dXdd9vqai=hGuQ8kuc9pgc9s8qqaq=dirpe0xb9q8qiLsFr0=vr0=vr0dc8meaabaqaciaacaGaaeqabaqabeGadaaakeaaiiGacqWFgpGzdaWgaaWcbaGaemyAaKgabeaakiabg2da9maaqafabaGae8NXdy2aaSbaaSqaaiabdMgaPjabcYcaSiabdQgaQbqabaaabaGaemOAaOgabeqdcqGHris5aOGaeiilaWcaaa@3ADA@

where the summation is over the set of AE sites which generate *informative *tags, *J*.

In the special case where all of the possible tags within a transcript are informative, the summation is from *j *= 1 to *k*_*i*_. Under this scenario, φi=1−(1−p)ki
 MathType@MTEF@5@5@+=feaafiart1ev1aaatCvAUfKttLearuWrP9MDH5MBPbIqV92AaeXatLxBI9gBaebbnrfifHhDYfgasaacH8akY=wiFfYdH8Gipec8Eeeu0xXdbba9frFj0=OqFfea0dXdd9vqai=hGuQ8kuc9pgc9s8qqaq=dirpe0xb9q8qiLsFr0=vr0=vr0dc8meaabaqaciaacaGaaeqabaqabeGadaaakeaaiiGacqWFgpGzdaWgaaWcbaGaemyAaKgabeaakiabg2da9iabigdaXiabgkHiTiabcIcaOiabigdaXiabgkHiTiabdchaWjabcMcaPmaaCaaaleqabaGaem4AaS2aaSbaaWqaaiabdMgaPbqabaaaaaaa@3AEC@. In general, the greater the number of AE sites that lead to informative tags |*J*|, the higher the tag formation probability *φ*_*i *_for a given gene. For a given set of AE sites, *φ*_*i *_increases with the cutting efficiency *p*, but the upper limit to *φ*_*i *_is limited by the number and position of uninformative tags. This is because as *p *approaches one, the probability of forming a tag from the 3' most or kith
 MathType@MTEF@5@5@+=feaafiart1ev1aaatCvAUfKttLearuWrP9MDH5MBPbIqV92AaeXatLxBI9gBaebbnrfifHhDYfgasaacH8akY=wiFfYdH8Gipec8Eeeu0xXdbba9frFj0=OqFfea0dXdd9vqai=hGuQ8kuc9pgc9s8qqaq=dirpe0xb9q8qiLsFr0=vr0=vr0dc8meaabaqaciaacaGaaeqabaqabeGadaaakeaacqWGRbWAdaqhaaWcbaGaemyAaKgabaGaeeiDaqNaeeiAaGgaaaaa@3259@ AE site also site approaches one. If *p *= 1 and the 3' most AE site leads to an informative tag, then *φ*_*i *_would be equal to one. In contrast, if the 3' most AE site leads to an ambiguous tag, then the *φ*_*i *_would equal zero. Thus, depending on the genome and the genes of interest, a high cutting efficiency may not only be unobtainable [18], it may also be undesirable.

Because transcripts come in discrete units, inference on the proportion of the *i*^th ^gene in the mRNA pool, *m*_*i*_, depends only on the sum of the tag frequencies in the tag pool for the *i*^th ^gene rather than the separate frequencies of each individual tag.

θi(m→)=miφiΦ¯
 MathType@MTEF@5@5@+=feaafiart1ev1aaatCvAUfKttLearuWrP9MDH5MBPbIqV92AaeXatLxBI9gBaebbnrfifHhDYfgasaacH8akY=wiFfYdH8Gipec8Eeeu0xXdbba9frFj0=OqFfea0dXdd9vqai=hGuQ8kuc9pgc9s8qqaq=dirpe0xb9q8qiLsFr0=vr0=vr0dc8meaabaqaciaacaGaaeqabaqabeGadaaakeaaiiGacqWF4oqCdaWgaaWcbaGaemyAaKgabeaakiabcIcaOiqbd2gaTzaalaGaeiykaKIaeyypa0JaemyBa02aaSbaaSqaaiabdMgaPbqabaGcdaWcaaqaaiab=z8aMnaaBaaaleaacqWGPbqAaeqaaaGcbaGafuOPdyKbaebaaaaaaa@3C02@

where

Φ¯=∑i=1nmiφi.
 MathType@MTEF@5@5@+=feaafiart1ev1aaatCvAUfKttLearuWrP9MDH5MBPbIqV92AaeXatLxBI9gBaebbnrfifHhDYfgasaacH8akY=wiFfYdH8Gipec8Eeeu0xXdbba9frFj0=OqFfea0dXdd9vqai=hGuQ8kuc9pgc9s8qqaq=dirpe0xb9q8qiLsFr0=vr0=vr0dc8meaabaqaciaacaGaaeqabaqabeGadaaakeaacuqHMoGrgaqeaiabg2da9maaqahabaGaemyBa02aaSbaaSqaaiabdMgaPbqabaacciGccqWFgpGzdaWgaaWcbaGaemyAaKgabeaaaeaacqWGPbqAcqGH9aqpcqaIXaqmaeaacqWGUbGBa0GaeyyeIuoakiabc6caUaaa@3D5A@

The term Φ¯
 MathType@MTEF@5@5@+=feaafiart1ev1aaatCvAUfKttLearuWrP9MDH5MBPbIqV92AaeXatLxBI9gBaebbnrfifHhDYfgasaacH8akY=wiFfYdH8Gipec8Eeeu0xXdbba9frFj0=OqFfea0dXdd9vqai=hGuQ8kuc9pgc9s8qqaq=dirpe0xb9q8qiLsFr0=vr0=vr0dc8meaabaqaciaacaGaaeqabaqabeGadaaakeaacuqHMoGrgaqeaaaa@2E3E@ denotes the mean tag formation probability where the contribution of a gene to its value is a function of both its mRNA frequency *m*_*i *_and tag formation *φ*_*i *_probability.

Intuitively, Eq. (3) states that the tag frequency for the *i*^th ^gene *θ*_*i *_is equal to its frequency in the mRNA pool weighted by the probability of tag being formed from its mRNA transcripts relative to the weighted average *φ *value for all of the mRNA genes that contribute to the tag pool. Incorporating the relative tag formation probability, *φ*_*i*_/Φ¯
 MathType@MTEF@5@5@+=feaafiart1ev1aaatCvAUfKttLearuWrP9MDH5MBPbIqV92AaeXatLxBI9gBaebbnrfifHhDYfgasaacH8akY=wiFfYdH8Gipec8Eeeu0xXdbba9frFj0=OqFfea0dXdd9vqai=hGuQ8kuc9pgc9s8qqaq=dirpe0xb9q8qiLsFr0=vr0=vr0dc8meaabaqaciaacaGaaeqabaqabeGadaaakeaacuqHMoGrgaqeaaaa@2E3E@ in Eq. (3), into inferences about *m*_*i *_is what distinguishes our approach from standard SAGE methods. Standard methods assume that *φ*_*i *_is constant across all genes and, therefore, *φ*_*i*_/Φ¯
 MathType@MTEF@5@5@+=feaafiart1ev1aaatCvAUfKttLearuWrP9MDH5MBPbIqV92AaeXatLxBI9gBaebbnrfifHhDYfgasaacH8akY=wiFfYdH8Gipec8Eeeu0xXdbba9frFj0=OqFfea0dXdd9vqai=hGuQ8kuc9pgc9s8qqaq=dirpe0xb9q8qiLsFr0=vr0=vr0dc8meaabaqaciaacaGaaeqabaqabeGadaaakeaacuqHMoGrgaqeaaaa@2E3E@ = 1. Hence, it equates the mRNA pool with the tag pool. However, because *φ*_*i *_varies between genes, this equality between pools does not hold.

#### Joint posterior distribution of m→
 MathType@MTEF@5@5@+=feaafiart1ev1aaatCvAUfKttLearuWrP9MDH5MBPbIqV92AaeXatLxBI9gBaebbnrfifHhDYfgasaacH8akY=wiFfYdH8Gipec8Eeeu0xXdbba9frFj0=OqFfea0dXdd9vqai=hGuQ8kuc9pgc9s8qqaq=dirpe0xb9q8qiLsFr0=vr0=vr0dc8meaabaqaciaacaGaaeqabaqabeGadaaakeaacuWGTbqBgaWcaaaa@2E21@

Using our definition of θ→
 MathType@MTEF@5@5@+=feaafiart1ev1aaatCvAUfKttLearuWrP9MDH5MBPbIqV92AaeXatLxBI9gBaebbnrfifHhDYfgasaacH8akY=wiFfYdH8Gipec8Eeeu0xXdbba9frFj0=OqFfea0dXdd9vqai=hGuQ8kuc9pgc9s8qqaq=dirpe0xb9q8qiLsFr0=vr0=vr0dc8meaabaqaciaacaGaaeqabaqabeGadaaakeaaiiGacuWF4oqCgaWcaaaa@2E7B@ and the assumption of a multinomial sampling distribution for the tags, it follows that the conditional probability of observing a sample of gene tags T→
 MathType@MTEF@5@5@+=feaafiart1ev1aaatCvAUfKttLearuWrP9MDH5MBPbIqV92AaeXatLxBI9gBaebbnrfifHhDYfgasaacH8akY=wiFfYdH8Gipec8Eeeu0xXdbba9frFj0=OqFfea0dXdd9vqai=hGuQ8kuc9pgc9s8qqaq=dirpe0xb9q8qiLsFr0=vr0=vr0dc8meaabaqaciaacaGaaeqabaqabeGadaaakeaacuWGubavgaWcaaaa@2DEF@ = {*T*_1_, *T*_2_, ... *T*_*n*_} given the gene expression levels m→
 MathType@MTEF@5@5@+=feaafiart1ev1aaatCvAUfKttLearuWrP9MDH5MBPbIqV92AaeXatLxBI9gBaebbnrfifHhDYfgasaacH8akY=wiFfYdH8Gipec8Eeeu0xXdbba9frFj0=OqFfea0dXdd9vqai=hGuQ8kuc9pgc9s8qqaq=dirpe0xb9q8qiLsFr0=vr0=vr0dc8meaabaqaciaacaGaaeqabaqabeGadaaakeaacuWGTbqBgaWcaaaa@2E21@ is,

P(T→|m→,φ→)=(T0T1,T2,...,Tn)∏i=1n(miφiΦ¯)Ti.
 MathType@MTEF@5@5@+=feaafiart1ev1aaatCvAUfKttLearuWrP9MDH5MBPbIqV92AaeXatLxBI9gBaebbnrfifHhDYfgasaacH8akY=wiFfYdH8Gipec8Eeeu0xXdbba9frFj0=OqFfea0dXdd9vqai=hGuQ8kuc9pgc9s8qqaq=dirpe0xb9q8qiLsFr0=vr0=vr0dc8meaabaqaciaacaGaaeqabaqabeGadaaakeaacqWGqbaucqGGOaakcuWGubavgaWcaiabcYha8jqbd2gaTzaalaGaeiilaWccciGaf8NXdyMbaSaacqGGPaqkcqGH9aqpdaqadaqaauaabeqaceaaaeaacqWGubavdaWgaaWcbaGaeGimaadabeaaaOqaaiabdsfaunaaBaaaleaacqaIXaqmaeqaaOGaeiilaWIaemivaq1aaSbaaSqaaiabikdaYaqabaGccqGGSaalcqGGUaGlcqGGUaGlcqGGUaGlcqGGSaalcqWGubavdaWgaaWcbaGaemOBa4gabeaaaaaakiaawIcacaGLPaaadaqeWbqaamaabmaabaWaaSaaaeaacqWGTbqBdaWgaaWcbaGaemyAaKgabeaakiab=z8aMnaaBaaaleaacqWGPbqAaeqaaaGcbaGafuOPdyKbaebaaaaacaGLOaGaayzkaaaaleaacqWGPbqAcqGH9aqpcqaIXaqmaeaacqWGUbGBa0Gaey4dIunakmaaCaaaleqabaGaemivaq1aaSbaaWqaaiabdMgaPbqabaaaaOGaeiOla4caaa@5C53@

Note that if the tagging probabilities *φ*_*i *_were equal for all genes, the joint distribution in Eq. (5) would simplify yielding P(T→|m→)=P(T→|θ→)
 MathType@MTEF@5@5@+=feaafiart1ev1aaatCvAUfKttLearuWrP9MDH5MBPbIqV92AaeXatLxBI9gBaebbnrfifHhDYfgasaacH8akY=wiFfYdH8Gipec8Eeeu0xXdbba9frFj0=OqFfea0dXdd9vqai=hGuQ8kuc9pgc9s8qqaq=dirpe0xb9q8qiLsFr0=vr0=vr0dc8meaabaqaciaacaGaaeqabaqabeGadaaakeaacqWGqbaucqGGOaakcuWGubavgaWcaiabcYha8jqbd2gaTzaalaGaeiykaKIaeyypa0JaemiuaaLaeiikaGIafmivaqLbaSaacqGG8baFiiGacuWF4oqCgaWcaiabcMcaPaaa@3C32@, i.e. the standard method for estimating gene expression levels.

We can also combine the above marginal probability distribution with a prior distribution *P*(*m*) to calculate the joint posterior distribution for m→
 MathType@MTEF@5@5@+=feaafiart1ev1aaatCvAUfKttLearuWrP9MDH5MBPbIqV92AaeXatLxBI9gBaebbnrfifHhDYfgasaacH8akY=wiFfYdH8Gipec8Eeeu0xXdbba9frFj0=OqFfea0dXdd9vqai=hGuQ8kuc9pgc9s8qqaq=dirpe0xb9q8qiLsFr0=vr0=vr0dc8meaabaqaciaacaGaaeqabaqabeGadaaakeaacuWGTbqBgaWcaaaa@2E21@ given the data T→
 MathType@MTEF@5@5@+=feaafiart1ev1aaatCvAUfKttLearuWrP9MDH5MBPbIqV92AaeXatLxBI9gBaebbnrfifHhDYfgasaacH8akY=wiFfYdH8Gipec8Eeeu0xXdbba9frFj0=OqFfea0dXdd9vqai=hGuQ8kuc9pgc9s8qqaq=dirpe0xb9q8qiLsFr0=vr0=vr0dc8meaabaqaciaacaGaaeqabaqabeGadaaakeaacuWGubavgaWcaaaa@2DEF@,

f(m→|T→,φ→)∝P(T→|m→,φ→)f(m→).
 MathType@MTEF@5@5@+=feaafiart1ev1aaatCvAUfKttLearuWrP9MDH5MBPbIqV92AaeXatLxBI9gBaebbnrfifHhDYfgasaacH8akY=wiFfYdH8Gipec8Eeeu0xXdbba9frFj0=OqFfea0dXdd9vqai=hGuQ8kuc9pgc9s8qqaq=dirpe0xb9q8qiLsFr0=vr0=vr0dc8meaabaqaciaacaGaaeqabaqabeGadaaakeaacqWGMbGzcqGGOaakcuWGTbqBgaWcaiabcYha8jqbdsfauzaalaGaeiilaWccciGaf8NXdyMbaSaacqGGPaqkcqGHDisTcqWGqbaucqGGOaakcuWGubavgaWcaiabcYha8jqbd2gaTzaalaGaeiilaWIaf8NXdyMbaSaacqGGPaqkcqWGMbGzcqGGOaakcuWGTbqBgaWcaiabcMcaPiabc6caUaaa@4736@

As in all Bayesian analyses, the choice of the prior distribution of m→
 MathType@MTEF@5@5@+=feaafiart1ev1aaatCvAUfKttLearuWrP9MDH5MBPbIqV92AaeXatLxBI9gBaebbnrfifHhDYfgasaacH8akY=wiFfYdH8Gipec8Eeeu0xXdbba9frFj0=OqFfea0dXdd9vqai=hGuQ8kuc9pgc9s8qqaq=dirpe0xb9q8qiLsFr0=vr0=vr0dc8meaabaqaciaacaGaaeqabaqabeGadaaakeaacuWGTbqBgaWcaaaa@2E21@ has some impact on the posterior distributions and the inferences based upon it. Given that m→
 MathType@MTEF@5@5@+=feaafiart1ev1aaatCvAUfKttLearuWrP9MDH5MBPbIqV92AaeXatLxBI9gBaebbnrfifHhDYfgasaacH8akY=wiFfYdH8Gipec8Eeeu0xXdbba9frFj0=OqFfea0dXdd9vqai=hGuQ8kuc9pgc9s8qqaq=dirpe0xb9q8qiLsFr0=vr0=vr0dc8meaabaqaciaacaGaaeqabaqabeGadaaakeaacuWGTbqBgaWcaaaa@2E21@ represents a set of frequencies, the Dirichlet distribution Dir(*α*_1_, *α*_2_, ... *α*_*n*_) is a logical prior distribution for m→
 MathType@MTEF@5@5@+=feaafiart1ev1aaatCvAUfKttLearuWrP9MDH5MBPbIqV92AaeXatLxBI9gBaebbnrfifHhDYfgasaacH8akY=wiFfYdH8Gipec8Eeeu0xXdbba9frFj0=OqFfea0dXdd9vqai=hGuQ8kuc9pgc9s8qqaq=dirpe0xb9q8qiLsFr0=vr0=vr0dc8meaabaqaciaacaGaaeqabaqabeGadaaakeaacuWGTbqBgaWcaaaa@2E21@. When *α*_*i *_= 1 for all genes, the prior distribution becomes a uniform or flat prior and eqn. (6) simplifies to,

f(m→|T→,φ→)∝∏i=1n(miφiΦ¯)Ti.
 MathType@MTEF@5@5@+=feaafiart1ev1aaatCvAUfKttLearuWrP9MDH5MBPbIqV92AaeXatLxBI9gBaebbnrfifHhDYfgasaacH8akY=wiFfYdH8Gipec8Eeeu0xXdbba9frFj0=OqFfea0dXdd9vqai=hGuQ8kuc9pgc9s8qqaq=dirpe0xb9q8qiLsFr0=vr0=vr0dc8meaabaqaciaacaGaaeqabaqabeGadaaakeaacqWGMbGzdaqadaqaaiqbd2gaTzaalaGaeiiFaWNafmivaqLbaSaacqGGSaaliiGacuWFgpGzgaWcaaGaayjkaiaawMcaaiabg2Hi1oaarahabaWaaeWaaeaadaWcaaqaaiabd2gaTnaaBaaaleaacqWGPbqAaeqaaOGae8NXdy2aaSbaaSqaaiabdMgaPbqabaaakeaacuqHMoGrgaqeaaaaaiaawIcacaGLPaaaaSqaaiabdMgaPjabg2da9iabigdaXaqaaiabd6gaUbqdcqGHpis1aOWaaWbaaSqabeaacqWGubavdaWgaaadbaGaemyAaKgabeaaaaGccqGGUaGlaaa@4C1D@

#### Joint posterior mode of m→
 MathType@MTEF@5@5@+=feaafiart1ev1aaatCvAUfKttLearuWrP9MDH5MBPbIqV92AaeXatLxBI9gBaebbnrfifHhDYfgasaacH8akY=wiFfYdH8Gipec8Eeeu0xXdbba9frFj0=OqFfea0dXdd9vqai=hGuQ8kuc9pgc9s8qqaq=dirpe0xb9q8qiLsFr0=vr0=vr0dc8meaabaqaciaacaGaaeqabaqabeGadaaakeaacuWGTbqBgaWcaaaa@2E21@

Using Bayes' Theorem eqn. (6) and employing a Lagrange multiplier to incorporate our constraints that ∑_*i *_*m*_*i *_= 1 and *m*_*i *_≥ 0, we generate the following implicit solution for the values of *m*_*i *_at the joint posterior mode, m^
 MathType@MTEF@5@5@+=feaafiart1ev1aaatCvAUfKttLearuWrP9MDH5MBPbIqV92AaeXatLxBI9gBaebbnrfifHhDYfgasaacH8akY=wiFfYdH8Gipec8Eeeu0xXdbba9frFj0=OqFfea0dXdd9vqai=hGuQ8kuc9pgc9s8qqaq=dirpe0xb9q8qiLsFr0=vr0=vr0dc8meaabaqaciaacaGaaeqabaqabeGadaaakeaacuWGTbqBgaqcaaaa@2E1F@,

m^i={0Ti+αi−1<0Ti+αi−1T0φiΦ¯^+α0−nelse
 MathType@MTEF@5@5@+=feaafiart1ev1aaatCvAUfKttLearuWrP9MDH5MBPbIqV92AaeXatLxBI9gBaebbnrfifHhDYfgasaacH8akY=wiFfYdH8Gipec8Eeeu0xXdbba9frFj0=OqFfea0dXdd9vqai=hGuQ8kuc9pgc9s8qqaq=dirpe0xb9q8qiLsFr0=vr0=vr0dc8meaabaqaciaacaGaaeqabaqabeGadaaakeaacuWGTbqBgaqcamaaBaaaleaacqWGPbqAaeqaaOGaeyypa0ZaaiqabeaafaqaaeGacaaabaGaeGimaadabaGaemivaq1aaSbaaSqaaiabdMgaPbqabaGccqGHRaWkiiGacqWFXoqydaWgaaWcbaGaemyAaKgabeaakiabgkHiTiabigdaXiabgYda8iabicdaWaqaamaalaaabaGaemivaq1aaSbaaSqaaiabdMgaPbqabaGccqGHRaWkcqWFXoqydaWgaaWcbaGaemyAaKgabeaakiabgkHiTiabigdaXaqaaiabdsfaunaaBaaaleaacqaIWaamaeqaaOWaaSaaaeaacqWFgpGzdaWgaaWcbaGaemyAaKgabeaaaOqaaiqbfA6agzaaryaajaaaaiabgUcaRiab=f7aHnaaBaaaleaacqaIWaamaeqaaOGaeyOeI0IaemOBa4gaaaqaaiabbwgaLjabbYgaSjabbohaZjabbwgaLbaaaiaawUhaaaaa@58DF@

Note that in the cases where the choice of the prior is such that *α*_*i *_< 1 and *T*_*i *_= 0 the mode of the posterior occurs at 0, the boundary of the parameter space. For convenience, we define *J *as the set of genes which satisfy the condition *T*_*i *_+ *α*_*i *_- 1 ≥ 0.

Although it might not be clear given our notation, the solution we present above is implicit since Φ¯^
 MathType@MTEF@5@5@+=feaafiart1ev1aaatCvAUfKttLearuWrP9MDH5MBPbIqV92AaeXatLxBI9gBaebbnrfifHhDYfgasaacH8akY=wiFfYdH8Gipec8Eeeu0xXdbba9frFj0=OqFfea0dXdd9vqai=hGuQ8kuc9pgc9s8qqaq=dirpe0xb9q8qiLsFr0=vr0=vr0dc8meaabaqaciaacaGaaeqabaqabeGadaaakeaacuqHMoGrgaqegaqcaaaa@2E4D@ depends on the set of m^
 MathType@MTEF@5@5@+=feaafiart1ev1aaatCvAUfKttLearuWrP9MDH5MBPbIqV92AaeXatLxBI9gBaebbnrfifHhDYfgasaacH8akY=wiFfYdH8Gipec8Eeeu0xXdbba9frFj0=OqFfea0dXdd9vqai=hGuQ8kuc9pgc9s8qqaq=dirpe0xb9q8qiLsFr0=vr0=vr0dc8meaabaqaciaacaGaaeqabaqabeGadaaakeaacuWGTbqBgaqcaaaa@2E1F@ values themselves. However, employing the constraint that the sum of m^
 MathType@MTEF@5@5@+=feaafiart1ev1aaatCvAUfKttLearuWrP9MDH5MBPbIqV92AaeXatLxBI9gBaebbnrfifHhDYfgasaacH8akY=wiFfYdH8Gipec8Eeeu0xXdbba9frFj0=OqFfea0dXdd9vqai=hGuQ8kuc9pgc9s8qqaq=dirpe0xb9q8qiLsFr0=vr0=vr0dc8meaabaqaciaacaGaaeqabaqabeGadaaakeaacuWGTbqBgaqcaaaa@2E1F@ values must equal one, we can generate the following implicit solution for Φ¯^
 MathType@MTEF@5@5@+=feaafiart1ev1aaatCvAUfKttLearuWrP9MDH5MBPbIqV92AaeXatLxBI9gBaebbnrfifHhDYfgasaacH8akY=wiFfYdH8Gipec8Eeeu0xXdbba9frFj0=OqFfea0dXdd9vqai=hGuQ8kuc9pgc9s8qqaq=dirpe0xb9q8qiLsFr0=vr0=vr0dc8meaabaqaciaacaGaaeqabaqabeGadaaakeaacuqHMoGrgaqegaqcaaaa@2E4D@,

1=∑i∈JTi+αi−1T0φiΦ¯^+α0−n.
 MathType@MTEF@5@5@+=feaafiart1ev1aaatCvAUfKttLearuWrP9MDH5MBPbIqV92AaeXatLxBI9gBaebbnrfifHhDYfgasaacH8akY=wiFfYdH8Gipec8Eeeu0xXdbba9frFj0=OqFfea0dXdd9vqai=hGuQ8kuc9pgc9s8qqaq=dirpe0xb9q8qiLsFr0=vr0=vr0dc8meaabaqaciaacaGaaeqabaqabeGadaaakeaacqaIXaqmcqGH9aqpdaaeqbqaamaalaaabaGaemivaq1aaSbaaSqaaiabdMgaPbqabaGccqGHRaWkiiGacqWFXoqydaWgaaWcbaGaemyAaKgabeaakiabgkHiTiabigdaXaqaaiabdsfaunaaBaaaleaacqaIWaamaeqaaOWaaSaaaeaacqWFgpGzdaWgaaWcbaGaemyAaKgabeaaaOqaaiqbfA6agzaaryaajaaaaiabgUcaRiab=f7aHnaaBaaaleaacqaIWaamaeqaaOGaeyOeI0IaemOBa4gaaaWcbaGaemyAaKMaeyicI4SaemOsaOeabeqdcqGHris5aOGaeiOla4caaa@4BB4@

Numerically solving equation (9) for Φ¯^
 MathType@MTEF@5@5@+=feaafiart1ev1aaatCvAUfKttLearuWrP9MDH5MBPbIqV92AaeXatLxBI9gBaebbnrfifHhDYfgasaacH8akY=wiFfYdH8Gipec8Eeeu0xXdbba9frFj0=OqFfea0dXdd9vqai=hGuQ8kuc9pgc9s8qqaq=dirpe0xb9q8qiLsFr0=vr0=vr0dc8meaabaqaciaacaGaaeqabaqabeGadaaakeaacuqHMoGrgaqegaqcaaaa@2E4D@ is straightforward and once done allows us to evaluate the solution for m^
 MathType@MTEF@5@5@+=feaafiart1ev1aaatCvAUfKttLearuWrP9MDH5MBPbIqV92AaeXatLxBI9gBaebbnrfifHhDYfgasaacH8akY=wiFfYdH8Gipec8Eeeu0xXdbba9frFj0=OqFfea0dXdd9vqai=hGuQ8kuc9pgc9s8qqaq=dirpe0xb9q8qiLsFr0=vr0=vr0dc8meaabaqaciaacaGaaeqabaqabeGadaaakeaacuWGTbqBgaqcaaaa@2E1F@ in eqn. (8) explicitly.

Under the uninformative, flat prior where *α*_*i *_= 1 for all genes, eqn. (9) can be solved explicitly and yields,

Φ¯^=T0∑i=1nTiφi
 MathType@MTEF@5@5@+=feaafiart1ev1aaatCvAUfKttLearuWrP9MDH5MBPbIqV92AaeXatLxBI9gBaebbnrfifHhDYfgasaacH8akY=wiFfYdH8Gipec8Eeeu0xXdbba9frFj0=OqFfea0dXdd9vqai=hGuQ8kuc9pgc9s8qqaq=dirpe0xb9q8qiLsFr0=vr0=vr0dc8meaabaqaciaacaGaaeqabaqabeGadaaakeaacuqHMoGrgaqegaqcaiabg2da9maalaaabaGaemivaq1aaSbaaSqaaiabicdaWaqabaaakeaadaaeWaqaamaalaaabaGaemivaq1aaSbaaSqaaiabdMgaPbqabaaakeaaiiGacqWFgpGzdaWgaaWcbaGaemyAaKgabeaaaaaabaGaemyAaKMaeyypa0JaeGymaedabaGaemOBa4ganiabggHiLdaaaaaa@3E7E@

and eqn. (8) simplifies to,

m^i=TiT0Φ¯^φi=Ti(∑j=1nTjφj)φi.
 MathType@MTEF@5@5@+=feaafiart1ev1aaatCvAUfKttLearuWrP9MDH5MBPbIqV92AaeXatLxBI9gBaebbnrfifHhDYfgasaacH8akY=wiFfYdH8Gipec8Eeeu0xXdbba9frFj0=OqFfea0dXdd9vqai=hGuQ8kuc9pgc9s8qqaq=dirpe0xb9q8qiLsFr0=vr0=vr0dc8meaabaqaciaacaGaaeqabaqabeGadaaakeaacuWGTbqBgaqcamaaBaaaleaacqWGPbqAaeqaaOGaeyypa0ZaaSaaaeaacqWGubavdaWgaaWcbaGaemyAaKgabeaaaOqaaiabdsfaunaaBaaaleaacqaIWaamaeqaaaaakmaalaaabaGafuOPdyKbaeHbaKaaaeaaiiGacqWFgpGzdaWgaaWcbaGaemyAaKgabeaaaaGccqGH9aqpdaWcaaqaaiabdsfaunaaBaaaleaacqWGPbqAaeqaaaGcbaWaaeWaaeaadaaeWaqaamaalaaabaGaemivaq1aaSbaaSqaaiabdQgaQbqabaaakeaacqWFgpGzdaWgaaWcbaGaemOAaOgabeaaaaaabaGaemOAaOMaeyypa0JaeGymaedabaGaemOBa4ganiabggHiLdaakiaawIcacaGLPaaacqWFgpGzdaWgaaWcbaGaemyAaKgabeaaaaGccqGGUaGlaaa@5133@

#### The marginal posterior distribution of *m*_*i*_

The multinomial conditional distribution and Dirichlet prior distributions used in Eq. (6) can be used to generate a marginal binomial distribution and prior beta distribution for the *i*th gene to yield the posterior distribution,

f(mi|T→,φ→,Φ¯)∝(miφiΦ¯)Ti(1−miφiΦ¯)T0−Timiα−1(1−mi)β−1,
 MathType@MTEF@5@5@+=feaafiart1ev1aaatCvAUfKttLearuWrP9MDH5MBPbIqV92AaeXatLxBI9gBaebbnrfifHhDYfgasaacH8akY=wiFfYdH8Gipec8Eeeu0xXdbba9frFj0=OqFfea0dXdd9vqai=hGuQ8kuc9pgc9s8qqaq=dirpe0xb9q8qiLsFr0=vr0=vr0dc8meaabaqaciaacaGaaeqabaqabeGadaaakeaacqWGMbGzdaqadaqaaiabd2gaTnaaBaaaleaacqWGPbqAaeqaaOWaaqqaaeaacuWGubavgaWcaiabcYcaSGGaciqb=z8aMzaalaGaeiilaWIafuOPdyKbaebaaiaawEa7aaGaayjkaiaawMcaaiabg2Hi1oaabmaabaWaaSaaaeaacqWGTbqBdaWgaaWcbaGaemyAaKgabeaakiab=z8aMnaaBaaaleaacqWGPbqAaeqaaaGcbaGafuOPdyKbaebaaaaacaGLOaGaayzkaaWaaWbaaSqabeaacqWGubavdaWgaaadbaGaemyAaKgabeaaaaGcdaqadaqaaiabigdaXiabgkHiTmaalaaabaGaemyBa02aaSbaaSqaaiabdMgaPbqabaGccqWFgpGzdaWgaaWcbaGaemyAaKgabeaaaOqaaiqbfA6agzaaraaaaaGaayjkaiaawMcaamaaCaaaleqabaGaemivaq1aaSbaaWqaaiabicdaWaqabaWccqGHsislcqWGubavdaWgaaadbaGaemyAaKgabeaaaaGccqWGTbqBdaqhaaWcbaGaemyAaKgabaGae8xSdeMaeyOeI0IaeGymaedaaOGaeiikaGIaeGymaeJaeyOeI0IaemyBa02aaSbaaSqaaiabdMgaPbqabaGccqGGPaqkdaahaaWcbeqaaiab=j7aIjabgkHiTiabigdaXaaakiabcYcaSaaa@6B41@

Where *α *= *α*_*i *_and β=∑j≠iαj
 MathType@MTEF@5@5@+=feaafiart1ev1aaatCvAUfKttLearuWrP9MDH5MBPbIqV92AaeXatLxBI9gBaebbnrfifHhDYfgasaacH8akY=wiFfYdH8Gipec8Eeeu0xXdbba9frFj0=OqFfea0dXdd9vqai=hGuQ8kuc9pgc9s8qqaq=dirpe0xb9q8qiLsFr0=vr0=vr0dc8meaabaqaciaacaGaaeqabaqabeGadaaakeaaiiGacqWFYoGycqGH9aqpdaaeqaqaaiab=f7aHnaaBaaaleaacqWGQbGAaeqaaaqaaiabdQgaQjabgcMi5kabdMgaPbqab0GaeyyeIuoaaaa@38D4@.

Because the marginal posterior distribution of *m*_*i*_, *f*(*m*_*i*_|T→
 MathType@MTEF@5@5@+=feaafiart1ev1aaatCvAUfKttLearuWrP9MDH5MBPbIqV92AaeXatLxBI9gBaebbnrfifHhDYfgasaacH8akY=wiFfYdH8Gipec8Eeeu0xXdbba9frFj0=OqFfea0dXdd9vqai=hGuQ8kuc9pgc9s8qqaq=dirpe0xb9q8qiLsFr0=vr0=vr0dc8meaabaqaciaacaGaaeqabaqabeGadaaakeaacuWGubavgaWcaaaa@2DEF@, φ→
 MathType@MTEF@5@5@+=feaafiart1ev1aaatCvAUfKttLearuWrP9MDH5MBPbIqV92AaeXatLxBI9gBaebbnrfifHhDYfgasaacH8akY=wiFfYdH8Gipec8Eeeu0xXdbba9frFj0=OqFfea0dXdd9vqai=hGuQ8kuc9pgc9s8qqaq=dirpe0xb9q8qiLsFr0=vr0=vr0dc8meaabaqaciaacaGaaeqabaqabeGadaaakeaaiiGacuWFgpGzgaWcaaaa@2E7E@), depends on the ratio of the focal gene *i*'s tag formation probability *φ*_*i *_relative to Φ¯
 MathType@MTEF@5@5@+=feaafiart1ev1aaatCvAUfKttLearuWrP9MDH5MBPbIqV92AaeXatLxBI9gBaebbnrfifHhDYfgasaacH8akY=wiFfYdH8Gipec8Eeeu0xXdbba9frFj0=OqFfea0dXdd9vqai=hGuQ8kuc9pgc9s8qqaq=dirpe0xb9q8qiLsFr0=vr0=vr0dc8meaabaqaciaacaGaaeqabaqabeGadaaakeaacuqHMoGrgaqeaaaa@2E3E@, the function implicitly depends on *m*_*i *_and the mRNA frequencies at all of the other genes besides *i*. In other words, Φ¯
 MathType@MTEF@5@5@+=feaafiart1ev1aaatCvAUfKttLearuWrP9MDH5MBPbIqV92AaeXatLxBI9gBaebbnrfifHhDYfgasaacH8akY=wiFfYdH8Gipec8Eeeu0xXdbba9frFj0=OqFfea0dXdd9vqai=hGuQ8kuc9pgc9s8qqaq=dirpe0xb9q8qiLsFr0=vr0=vr0dc8meaabaqaciaacaGaaeqabaqabeGadaaakeaacuqHMoGrgaqeaaaa@2E3E@ is a function of and, therefore technically changes with, *m*_*i*_. These changes in Φ¯
 MathType@MTEF@5@5@+=feaafiart1ev1aaatCvAUfKttLearuWrP9MDH5MBPbIqV92AaeXatLxBI9gBaebbnrfifHhDYfgasaacH8akY=wiFfYdH8Gipec8Eeeu0xXdbba9frFj0=OqFfea0dXdd9vqai=hGuQ8kuc9pgc9s8qqaq=dirpe0xb9q8qiLsFr0=vr0=vr0dc8meaabaqaciaacaGaaeqabaqabeGadaaakeaacuqHMoGrgaqeaaaa@2E3E@ with *m*_*i *_can be taken into account when evaluating Eq. (12) by re-estimating Φ¯
 MathType@MTEF@5@5@+=feaafiart1ev1aaatCvAUfKttLearuWrP9MDH5MBPbIqV92AaeXatLxBI9gBaebbnrfifHhDYfgasaacH8akY=wiFfYdH8Gipec8Eeeu0xXdbba9frFj0=OqFfea0dXdd9vqai=hGuQ8kuc9pgc9s8qqaq=dirpe0xb9q8qiLsFr0=vr0=vr0dc8meaabaqaciaacaGaaeqabaqabeGadaaakeaacuqHMoGrgaqeaaaa@2E3E@ given a specific value of *m*_*i*_. Reestimating Φ¯
 MathType@MTEF@5@5@+=feaafiart1ev1aaatCvAUfKttLearuWrP9MDH5MBPbIqV92AaeXatLxBI9gBaebbnrfifHhDYfgasaacH8akY=wiFfYdH8Gipec8Eeeu0xXdbba9frFj0=OqFfea0dXdd9vqai=hGuQ8kuc9pgc9s8qqaq=dirpe0xb9q8qiLsFr0=vr0=vr0dc8meaabaqaciaacaGaaeqabaqabeGadaaakeaacuqHMoGrgaqeaaaa@2E3E@ is, however, numerically intensive. Further, a majority of the probability mass of the marginal distribution occurs in the region very close to Φ¯
 MathType@MTEF@5@5@+=feaafiart1ev1aaatCvAUfKttLearuWrP9MDH5MBPbIqV92AaeXatLxBI9gBaebbnrfifHhDYfgasaacH8akY=wiFfYdH8Gipec8Eeeu0xXdbba9frFj0=OqFfea0dXdd9vqai=hGuQ8kuc9pgc9s8qqaq=dirpe0xb9q8qiLsFr0=vr0=vr0dc8meaabaqaciaacaGaaeqabaqabeGadaaakeaacuqHMoGrgaqeaaaa@2E3E@ (as estimated in eqn. (8)). Thus, the changes in Φ¯
 MathType@MTEF@5@5@+=feaafiart1ev1aaatCvAUfKttLearuWrP9MDH5MBPbIqV92AaeXatLxBI9gBaebbnrfifHhDYfgasaacH8akY=wiFfYdH8Gipec8Eeeu0xXdbba9frFj0=OqFfea0dXdd9vqai=hGuQ8kuc9pgc9s8qqaq=dirpe0xb9q8qiLsFr0=vr0=vr0dc8meaabaqaciaacaGaaeqabaqabeGadaaakeaacuqHMoGrgaqeaaaa@2E3E@ over the most probable values of *m*_*i *_are negligable and, consequently, the effect of these changes on the marginal distribution of *m*_*i *_is also negligable. As a result, we ignore any impact changing *m*_*i *_might have on our estimate of Φ¯
 MathType@MTEF@5@5@+=feaafiart1ev1aaatCvAUfKttLearuWrP9MDH5MBPbIqV92AaeXatLxBI9gBaebbnrfifHhDYfgasaacH8akY=wiFfYdH8Gipec8Eeeu0xXdbba9frFj0=OqFfea0dXdd9vqai=hGuQ8kuc9pgc9s8qqaq=dirpe0xb9q8qiLsFr0=vr0=vr0dc8meaabaqaciaacaGaaeqabaqabeGadaaakeaacuqHMoGrgaqeaaaa@2E3E@ and, instead, treat Φ¯
 MathType@MTEF@5@5@+=feaafiart1ev1aaatCvAUfKttLearuWrP9MDH5MBPbIqV92AaeXatLxBI9gBaebbnrfifHhDYfgasaacH8akY=wiFfYdH8Gipec8Eeeu0xXdbba9frFj0=OqFfea0dXdd9vqai=hGuQ8kuc9pgc9s8qqaq=dirpe0xb9q8qiLsFr0=vr0=vr0dc8meaabaqaciaacaGaaeqabaqabeGadaaakeaacuqHMoGrgaqeaaaa@2E3E@ as a constant (i.e. Φ¯=Φ¯^
 MathType@MTEF@5@5@+=feaafiart1ev1aaatCvAUfKttLearuWrP9MDH5MBPbIqV92AaeXatLxBI9gBaebbnrfifHhDYfgasaacH8akY=wiFfYdH8Gipec8Eeeu0xXdbba9frFj0=OqFfea0dXdd9vqai=hGuQ8kuc9pgc9s8qqaq=dirpe0xb9q8qiLsFr0=vr0=vr0dc8meaabaqaciaacaGaaeqabaqabeGadaaakeaacuqHMoGrgaqeaiabg2da9iqbfA6agzaaryaajaaaaa@30E5@) in the calculations that follow.

#### Approximations of the marginal posterior mode of *m*_*i*_

Ignoring the dependence of Φ¯
 MathType@MTEF@5@5@+=feaafiart1ev1aaatCvAUfKttLearuWrP9MDH5MBPbIqV92AaeXatLxBI9gBaebbnrfifHhDYfgasaacH8akY=wiFfYdH8Gipec8Eeeu0xXdbba9frFj0=OqFfea0dXdd9vqai=hGuQ8kuc9pgc9s8qqaq=dirpe0xb9q8qiLsFr0=vr0=vr0dc8meaabaqaciaacaGaaeqabaqabeGadaaakeaacuqHMoGrgaqeaaaa@2E3E@ on *m*_*i*_, we differentiate the marginal probability of *m*_*i *_in eqn. (12) with respect to *m*_*i *_and set it equal to zero. This results in a quadratic solution for the marginal mode of *m*_*i*_, m˜
 MathType@MTEF@5@5@+=feaafiart1ev1aaatCvAUfKttLearuWrP9MDH5MBPbIqV92AaeXatLxBI9gBaebbnrfifHhDYfgasaacH8akY=wiFfYdH8Gipec8Eeeu0xXdbba9frFj0=OqFfea0dXdd9vqai=hGuQ8kuc9pgc9s8qqaq=dirpe0xb9q8qiLsFr0=vr0=vr0dc8meaabaqaciaacaGaaeqabaqabeGadaaakeaacuWGTbqBgaacaaaa@2E1E@. The quadratic solution, however, is quite complex and we present it in Appendix B. There we also derive a much simpler approximation based on a first order Taylor series to expansion which gives,

m˜i≈max⁡(0,Ti+α−1φiΦ¯^(T0−(Ti+α−1))+(Ti+α−1+β−1)).
 MathType@MTEF@5@5@+=feaafiart1ev1aaatCvAUfKttLearuWrP9MDH5MBPbIqV92AaeXatLxBI9gBaebbnrfifHhDYfgasaacH8akY=wiFfYdH8Gipec8Eeeu0xXdbba9frFj0=OqFfea0dXdd9vqai=hGuQ8kuc9pgc9s8qqaq=dirpe0xb9q8qiLsFr0=vr0=vr0dc8meaabaqaciaacaGaaeqabaqabeGadaaakeaacuWGTbqBgaacamaaBaaaleaacqWGPbqAaeqaaOGaeyisISRagiyBa0MaeiyyaeMaeiiEaG3aaeWaaeaacqaIWaamcqGGSaaldaWcaaqaaiabdsfaunaaBaaaleaacqWGPbqAaeqaaOGaey4kaSccciGae8xSdeMaeyOeI0IaeGymaedabaWaaSaaaeaacqWFgpGzdaWgaaWcbaGaemyAaKgabeaaaOqaaiqbfA6agzaaryaajaaaaiabcIcaOiabdsfaunaaBaaaleaacqaIWaamaeqaaOGaeyOeI0IaeiikaGIaemivaq1aaSbaaSqaaiabdMgaPbqabaGccqGHRaWkcqWFXoqycqGHsislcqaIXaqmcqGGPaqkcqGGPaqkcqGHRaWkcqGGOaakcqWGubavdaWgaaWcbaGaemyAaKgabeaakiabgUcaRiab=f7aHjabgkHiTiabigdaXiabgUcaRiab=j7aIjabgkHiTiabigdaXiabcMcaPaaaaiaawIcacaGLPaaacqGGUaGlaaa@61B9@

This solution for the mode can be simplified further depending on the specific assumptions made about *α *and *β*.

For example, in calculating the marginal posterior distribution of *m*_*i *_under a uniform prior, the joint Dirichlet prior with *α*_*j *_= 1 for all genes reduces to the parameters *α*_*i *_= 1 and *β*_*i *_= *n *- 1 for the marginal prior distribution distribution. With these parameter values,



assuming that *n *is large relative to *T*_*i *_and 1.

If (*n/T*_0_) × (Φ¯
 MathType@MTEF@5@5@+=feaafiart1ev1aaatCvAUfKttLearuWrP9MDH5MBPbIqV92AaeXatLxBI9gBaebbnrfifHhDYfgasaacH8akY=wiFfYdH8Gipec8Eeeu0xXdbba9frFj0=OqFfea0dXdd9vqai=hGuQ8kuc9pgc9s8qqaq=dirpe0xb9q8qiLsFr0=vr0=vr0dc8meaabaqaciaacaGaaeqabaqabeGadaaakeaacuqHMoGrgaqeaaaa@2E3E@/*φ*_*i*_) ≪ 1, then we can take another first order Taylor series approximation around this term at zero to get a solution for the marginal mode as a function of the joint mode,

m˜i≈m^i(1−nT0Φ¯^φj)
 MathType@MTEF@5@5@+=feaafiart1ev1aaatCvAUfKttLearuWrP9MDH5MBPbIqV92AaeXatLxBI9gBaebbnrfifHhDYfgasaacH8akY=wiFfYdH8Gipec8Eeeu0xXdbba9frFj0=OqFfea0dXdd9vqai=hGuQ8kuc9pgc9s8qqaq=dirpe0xb9q8qiLsFr0=vr0=vr0dc8meaabaqaciaacaGaaeqabaqabeGadaaakeaacuWGTbqBgaacamaaBaaaleaacqWGPbqAaeqaaOGaeyisISRafmyBa0MbaKaadaWgaaWcbaGaemyAaKgabeaakmaabmaabaGaeGymaeJaeyOeI0YaaSaaaeaacqWGUbGBaeaacqWGubavdaWgaaWcbaGaeGimaadabeaaaaGcdaWcaaqaaiqbfA6agzaaryaajaaabaacciGae8NXdy2aaSbaaSqaaiabdQgaQbqabaaaaaGccaGLOaGaayzkaaaaaa@4098@

Thus we see that the marginal mode of *m*_*i *_is always less than the value at the joint mode with the prior *α*_*i *_= 1 for all genes.

Alternatively, if we assume an alternative prior of *α*_*i *_= 1/*n*, which implies that *β*_*i *_= 1 - 1/*n*, then using the same assumptions and approach as before we obtain the following set of approximate marginal modes,

m˜i≈{0Ti=0Φ¯^φi1nT0Ti=1Φ¯^φiTi−1T0Ti≥2.
 MathType@MTEF@5@5@+=feaafiart1ev1aqatCvAUfKttLearuWrP9MDH5MBPbIqV92AaeXatLxBI9gBaebbnrfifHhDYfgasaacH8akY=wiFfYdH8Gipec8Eeeu0xXdbba9frFj0=OqFfea0dXdd9vqai=hGuQ8kuc9pgc9s8qqaq=dirpe0xb9q8qiLsFr0=vr0=vr0dc8meaabaqaciaacaGaaeqabaqabeGadaaakeaacuWGTbqBgaacamaaBaaaleaacqWGPbqAaeqaaOGaeyisIS7aaiqabeaafaqaaeWacaaabaGaeGimaadabaGaemivaq1aaSbaaSqaaiabdMgaPbqabaGccqGH9aqpcqaIWaamaeaadaWcaaqaaiqbfA6agzaaryaajaaabaacciGae8NXdy2aaSbaaSqaaiabdMgaPbqabaaaaOWaaSaaaeaacqaIXaqmaeaacqWGUbGBcqWGubavdaWgaaWcbaGaeGimaadabeaaaaaakeaacqWGubavdaWgaaWcbaGaemyAaKgabeaakiabg2da9iabigdaXaqaamaalaaabaGafuOPdyKbaeHbaKaaaeaacqWFgpGzdaWgaaWcbaGaemyAaKgabeaaaaGcdaWcaaqaaiabdsfaunaaBaaaleaacqWGPbqAaeqaaOGaeyOeI0IaeGymaedabaGaemivaq1aaSbaaSqaaiabicdaWaqabaaaaaGcbaGaemivaq1aaSbaaSqaaiabdMgaPbqabaGccqGHLjYScqaIYaGmcqGGUaGlaaaacaGL7baaaaa@58FE@

As a final point of comparison we compare our Bayesian approaches in which we impose a consistent set of prior values on the probabilities of m→
 MathType@MTEF@5@5@+=feaafiart1ev1aaatCvAUfKttLearuWrP9MDH5MBPbIqV92AaeXatLxBI9gBaebbnrfifHhDYfgasaacH8akY=wiFfYdH8Gipec8Eeeu0xXdbba9frFj0=OqFfea0dXdd9vqai=hGuQ8kuc9pgc9s8qqaq=dirpe0xb9q8qiLsFr0=vr0=vr0dc8meaabaqaciaacaGaaeqabaqabeGadaaakeaacuWGTbqBgaWcaaaa@2E21@ with a likelihood based approach. Estimating *m*_*i *_based on likelihood is equivalent to the beta parameters *α*_*i *_= 1 and *β*_*i *_= 1,

m˜i=Φ¯φiTiT0.
 MathType@MTEF@5@5@+=feaafiart1ev1aaatCvAUfKttLearuWrP9MDH5MBPbIqV92AaeXatLxBI9gBaebbnrfifHhDYfgasaacH8akY=wiFfYdH8Gipec8Eeeu0xXdbba9frFj0=OqFfea0dXdd9vqai=hGuQ8kuc9pgc9s8qqaq=dirpe0xb9q8qiLsFr0=vr0=vr0dc8meaabaqaciaacaGaaeqabaqabeGadaaakeaacuWGTbqBgaacamaaBaaaleaacqWGPbqAaeqaaOGaeyypa0ZaaSaaaeaacuqHMoGrgaqeaaqaaGGaciab=z8aMnaaBaaaleaacqWGPbqAaeqaaaaakmaalaaabaGaemivaq1aaSbaaSqaaiabdMgaPbqabaaakeaacqWGubavdaWgaaWcbaGaeGimaadabeaaaaGccqGGUaGlaaa@3BB3@

Note that although this solution is equivalent to the value of *m*_*i *_at the joint mode with a flat prior, it is not actually consistent with that model. This is because the maximum likelihood parameters imply that *α *= *α*_*i *_= 1 for the *i*th gene and yet the sum of the prior parameters for the remaining *n *- 1 genes is 1 rather than *n *- 1.

### Model application and validation

#### SAGE data and observed tag counts *T*_*i*_

In the data set provided by Velculescu *et al*. 1997, NlaIII is the Anchoring Enzyme whose recognition sequence is 5'-CATG-3'. BsmFI is the Tagging Enzyme, which gives 14-bp tags. Uninformative tags (i.e. tags which could have come from multiple genes) were excluded from the calculation of *φ*_*i *_and from our tag counts *T*_*i*_. As indicated earlier, the tag counts for an individual gene *T*_*i *_is equal to the sum of all informative tags observed for the *i*^th ^gene in a given experiment. This exclusion of counts for ambiguous tags is also employed in other SAGE analyses.

#### Estimation of cutting efficiency *p*

**Figure 2:**
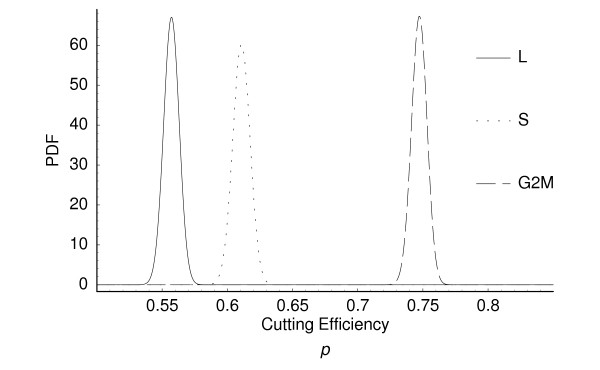
Posterior probability distributions for the AE cutting effciencies from three different SAGE experiments dicussed in [9]. The experiments were performed with cells at either log growth (L), S-phase arrested (S) or differ G2M-phase arrested. Distributions were generated as in Appendix A. The posterior modes and 95% confidence intervals are provided in Table 2.

We applied our method to a SAGE data set generated in *Saccharomyces cerevisiae *[9]. The first step during the implementation is to calculate the average cleavage efficiency of AE, *p*. The distribution of tags within the coding sequence (CDS) is a function of *p*. For example if *p *is close to one, then we expect to find the final intra-CDS tag to represent the vast majority of tags seen. Conversely if *p *is small, then the expected frequency of tags will increase little with position. In Appendix A we show how cleavage efficiency parameter *p *can be estimated from the observed distribution of intra-CDS tags from multiple genes using a Bayesian approach. The posterior mode and 95% PI for the L, S, and G2M phases are presented in Table 2 and the distributions are illustrated in Figure 2. Despite being carried out in the same lab and, presumably, under similar conditions, our analysis indicates that there is significant variation in cleavage effciencies between experiments.

**Table 2:** Parameter Estimates

Experimental Treatment			
Variables	L	S	G2/M
*P*	0.577 (0.545, 0.569)	0.61 (0.597,0.623)	0.748 (0.735, 0.758)
Joint Posterior Mode Estimate: Φ¯ MathType@MTEF@5@5@+=feaafiart1ev1aaatCvAUfKttLearuWrP9MDH5MBPbIqV92AaeXatLxBI9gBaebbnrfifHhDYfgasaacH8akY=wiFfYdH8Gipec8Eeeu0xXdbba9frFj0=OqFfea0dXdd9vqai=hGuQ8kuc9pgc9s8qqaq=dirpe0xb9q8qiLsFr0=vr0=vr0dc8meaabaqaciaacaGaaeqabaqabeGadaaakeaacuqHMoGrgaqeaaaa@2E3E@	0.764	0.797	0.862
Simulation Based Estimate: Φ¯ MathType@MTEF@5@5@+=feaafiart1ev1aaatCvAUfKttLearuWrP9MDH5MBPbIqV92AaeXatLxBI9gBaebbnrfifHhDYfgasaacH8akY=wiFfYdH8Gipec8Eeeu0xXdbba9frFj0=OqFfea0dXdd9vqai=hGuQ8kuc9pgc9s8qqaq=dirpe0xb9q8qiLsFr0=vr0=vr0dc8meaabaqaciaacaGaaeqabaqabeGadaaakeaacuqHMoGrgaqeaaaa@2E3E@	0.777 (0.773, 0.781)	0.806 (0.802,0.809)	0.861 (0.857,0.865)

Posterior mode and 95%PI values for the AE cleavage efficency *p*, posterior mode value for the mean tag formation probability Φ¯^
 MathType@MTEF@5@5@+=feaafiart1ev1aaatCvAUfKttLearuWrP9MDH5MBPbIqV92AaeXatLxBI9gBaebbnrfifHhDYfgasaacH8akY=wiFfYdH8Gipec8Eeeu0xXdbba9frFj0=OqFfea0dXdd9vqai=hGuQ8kuc9pgc9s8qqaq=dirpe0xb9q8qiLsFr0=vr0=vr0dc8meaabaqaciaacaGaaeqabaqabeGadaaakeaacuqHMoGrgaqegaqcaaaa@2E4D@, and simulation based estimates for Φ¯¯
 MathType@MTEF@5@5@+=feaafiart1ev1aaatCvAUfKttLearuWrP9MDH5MBPbIqV92AaeXatLxBI9gBaebbnrfifHhDYfgasaacH8akY=wiFfYdH8Gipec8Eeeu0xXdbba9frFj0=OqFfea0dXdd9vqai=hGuQ8kuc9pgc9s8qqaq=dirpe0xb9q8qiLsFr0=vr0=vr0dc8meaabaqaciaacaGaaeqabaqabeGadaaakeaacuqHMoGrgaqegaqeaaaa@2E55@ for the three experiments in [9]: log growth (L), S phase-arrest, and G2/M phase-arrest. Numbers in parentheses are the lower and upper bounds of 95% PI. Parameters for the Dirichlet prior distribution on m→
 MathType@MTEF@5@5@+=feaafiart1ev1aaatCvAUfKttLearuWrP9MDH5MBPbIqV92AaeXatLxBI9gBaebbnrfifHhDYfgasaacH8akY=wiFfYdH8Gipec8Eeeu0xXdbba9frFj0=OqFfea0dXdd9vqai=hGuQ8kuc9pgc9s8qqaq=dirpe0xb9q8qiLsFr0=vr0=vr0dc8meaabaqaciaacaGaaeqabaqabeGadaaakeaacuWGTbqBgaWcaaaa@2E21@ was *α*_*i *_= 1 for all genes.

#### Calculation of tag formation probability *φ*

The calculation *φ*_*i *_is, in part, based on the number of AE sites within an mRNA transcript. As a result, we need to know the transcript boundaries for every gene in order to determine all the possible AE sites for each gene. For 2342 genes we obtained the transcript boundaries from the tiling array data set [19]. With this subset of genes we also calculated the median 5' and 3' UTRs and used these values, 70 bp and 95 bp, respectively, for the remaining genes. We then inferred all potential AE sites for every transcript.

Having an estimate of *p *and knowing the set of possible informative tag sites within each gene makes it possible to calculate the tag formation probability *φ*_*i*_. For each individual gene, we used eqns. (1) and (2) and the posterior mode of *p *for a given experiment. Thus, because *p *varies between experiments, *φ*_*i *_also varies between experiments.

**Figure 3:**
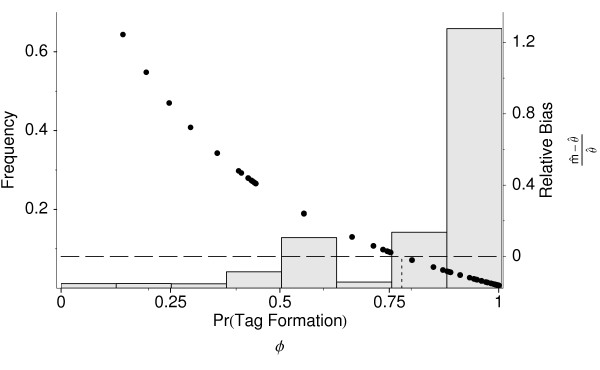
Composite diagram of tag formation probabilities *φ *and adjustment of mRNA estimates due to the tagging process for the Log Phase experiment in [9]. Histogram of the relative frequencies of tag formation probabilities *φ*_*i *_for the *Saccharomyces cerevisiae *genome during log growth phase and the corresponding scaling. Histogram scale is indicated on the left axis. Tag formation probabilities were calculated using eqns. (1) and (2) with the cutting efficiency parameter set to the posterior mode for this experiment, i.e. *p *= 0.56. The relative difference between the adjusted and standard mRNA estimates, (m^−θ^)/θ^
 MathType@MTEF@5@5@+=feaafiart1ev1aaatCvAUfKttLearuWrP9MDH5MBPbIqV92AaeXatLxBI9gBaebbnrfifHhDYfgasaacH8akY=wiFfYdH8Gipec8Eeeu0xXdbba9frFj0=OqFfea0dXdd9vqai=hGuQ8kuc9pgc9s8qqaq=dirpe0xb9q8qiLsFr0=vr0=vr0dc8meaabaqaciaacaGaaeqabaqabeGadaaakeaacqGGOaakcuWGTbqBgaqcaiabgkHiTGGaciqb=H7aXzaajaGaeiykaKIaei4la8Iaf8hUdeNbaKaaaaa@3532@, for each gene is plotted relative to the right axis and indicated with a •.

The distribution of tag formation probabilities for the log phase *L *experiment and its scaling effect on mRNA frequency inferences are illustrated in Figure 3. Note that intergenic variation in tag formation probabilities *φ*_*i *_vary from zero to one. Because genes with *φ*_*i *_= 0 are invisible with respect to SAGE experiments, they have been excluded from the figure. Intergenic variation in *φ*_*i *_appears to be bimodal with a peak around 0.56 and 0.9. The first peak in the distribution of *φ*_*i *_corresponds to genes with a single, unambiguous tag. The second peak in the distribution corresponds to genes with multiple unambiguous tags. Estimates of *φ *for all three experiments (L, S, G2M) as well as unambiguous tag counts and frequencies at the joint mode can be found in Additional Files 7, 8, 9.

#### Posterior distributions and statistics

For the following calculations we worked only with genes in *Saccharomyces cerevisiae *with a tag formation probability greater than 10^-7^. This non-zero cut off prevented us from including the genes where an observed tag is most likely due to an experimental errors rather than coming from the gene itself. Our dataset consisted of 6069 genes and we assumed a flat, uninformative prior of *α*_*i *_= 1 for all genes in the analysis presented here.

**Figure 4:**
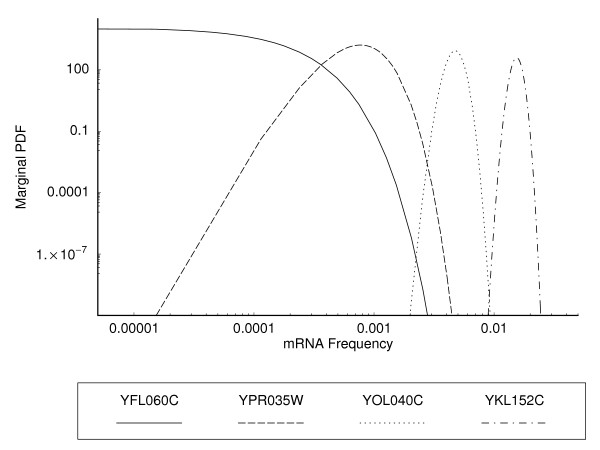
Examples of posterior marginal probability distributions for four genes, YFL060C, YPR035W, YOL040C, and YKL152C, during log phase based on data in [9]. Genes were chosen to cover a wide range of tag formation probabilities *φ*_*i *_and counts *T*_*i*_. More specifically, these genes had tag formation probabilities *φ*_*i *_of 0.356879, 0.44494, 0.98255, and 0.555, respectively, and observed tags counts of 0, 10, 103, and 228, respectively.

**Figure 5:**
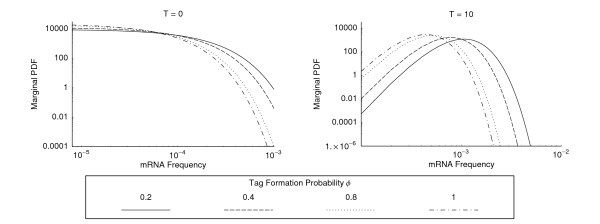
Illustration of how changing the tag formation probability *φ *affects the posterior marginal distributions under two different scenarios: (a) when no tags are observed for a particular gene and (b) when ten tags are observed for a particular gene. In (a) where no tags are observed, the posterior mode occurs on the boundary of the parameter space and changing *φ *has no effect on the mode. Increasing *φ *does, however, decrease the width of the distribution. In (b) where ten tags are observed, increasing *φ *leads to a decrease in the mode and also decreases the absolute width of the distribution (which is indicated on the log scale by shifting to the left).

With this set of genes we calculated the joint modes of the posterior distributions using eqn. (8) and the posterior marginal distributions (12) numerically. Examples of these marginal distributions for four specific genes are illustrated in Figure 4. Summaries of the posterior marginal distributions for all genes can be found in Additional Files 1, 2, 3. The more general effect that the tag formation probability and the number of tag counts have on the marginal distributions are illustrated in Figure 5.

We found the marginal mode numerically by maximizing (12). For each of these genes we also used (12) to calculate the posterior 95% probability intervals (PI). When comparing our numerical maximization of the marginal distribution to our various approximations, we find them to generally be withing a factor of 10^-5^

In order to verify the accuracy of the above results in a more independent manner, we simulated the joint posterior distribution utilizing a Gibbs Sampling strategies as discussed in [20]. From this joint posterior distribution we can obtain the appropriate marginal distributions. We find that our estimates of the means of the numerical and simulation based posterior marginal distributions are in good agreement.

Although we can calculate the the joint mode estimate of Φ¯
 MathType@MTEF@5@5@+=feaafiart1ev1aaatCvAUfKttLearuWrP9MDH5MBPbIqV92AaeXatLxBI9gBaebbnrfifHhDYfgasaacH8akY=wiFfYdH8Gipec8Eeeu0xXdbba9frFj0=OqFfea0dXdd9vqai=hGuQ8kuc9pgc9s8qqaq=dirpe0xb9q8qiLsFr0=vr0=vr0dc8meaabaqaciaacaGaaeqabaqabeGadaaakeaacuqHMoGrgaqeaaaa@2E3E@ directly, we cannot easily estimate its 95% PI. Instead we calculated Φ¯
 MathType@MTEF@5@5@+=feaafiart1ev1aaatCvAUfKttLearuWrP9MDH5MBPbIqV92AaeXatLxBI9gBaebbnrfifHhDYfgasaacH8akY=wiFfYdH8Gipec8Eeeu0xXdbba9frFj0=OqFfea0dXdd9vqai=hGuQ8kuc9pgc9s8qqaq=dirpe0xb9q8qiLsFr0=vr0=vr0dc8meaabaqaciaacaGaaeqabaqabeGadaaakeaacuqHMoGrgaqeaaaa@2E3E@ for each of our simulations and used these values to evaluate our uncertainty in Φ¯
 MathType@MTEF@5@5@+=feaafiart1ev1aaatCvAUfKttLearuWrP9MDH5MBPbIqV92AaeXatLxBI9gBaebbnrfifHhDYfgasaacH8akY=wiFfYdH8Gipec8Eeeu0xXdbba9frFj0=OqFfea0dXdd9vqai=hGuQ8kuc9pgc9s8qqaq=dirpe0xb9q8qiLsFr0=vr0=vr0dc8meaabaqaciaacaGaaeqabaqabeGadaaakeaacuqHMoGrgaqeaaaa@2E3E@. The results are presented in Table 2 and they indicate that Φ¯
 MathType@MTEF@5@5@+=feaafiart1ev1aaatCvAUfKttLearuWrP9MDH5MBPbIqV92AaeXatLxBI9gBaebbnrfifHhDYfgasaacH8akY=wiFfYdH8Gipec8Eeeu0xXdbba9frFj0=OqFfea0dXdd9vqai=hGuQ8kuc9pgc9s8qqaq=dirpe0xb9q8qiLsFr0=vr0=vr0dc8meaabaqaciaacaGaaeqabaqabeGadaaakeaacuqHMoGrgaqeaaaa@2E3E@ can varies significantly between the experimental treatments, reflecting signficant changes in the set of genes contributing to the mRNA pool.

Somewhat surprisingly, we find that in two of the three cases the joint mode estimate of Φ¯
 MathType@MTEF@5@5@+=feaafiart1ev1aaatCvAUfKttLearuWrP9MDH5MBPbIqV92AaeXatLxBI9gBaebbnrfifHhDYfgasaacH8akY=wiFfYdH8Gipec8Eeeu0xXdbba9frFj0=OqFfea0dXdd9vqai=hGuQ8kuc9pgc9s8qqaq=dirpe0xb9q8qiLsFr0=vr0=vr0dc8meaabaqaciaacaGaaeqabaqabeGadaaakeaacuqHMoGrgaqeaaaa@2E3E@ does not overlap with the 95% PI of Φ¯
 MathType@MTEF@5@5@+=feaafiart1ev1aaatCvAUfKttLearuWrP9MDH5MBPbIqV92AaeXatLxBI9gBaebbnrfifHhDYfgasaacH8akY=wiFfYdH8Gipec8Eeeu0xXdbba9frFj0=OqFfea0dXdd9vqai=hGuQ8kuc9pgc9s8qqaq=dirpe0xb9q8qiLsFr0=vr0=vr0dc8meaabaqaciaacaGaaeqabaqabeGadaaakeaacuqHMoGrgaqeaaaa@2E3E@ based on our simulations. This apparent paradox can be explained by the fact that the tag formation probability of a gene is not independent of its mRNA expression level. More specifically, genes with low expression levels are more likely to be longer and have unique tags than genes with high expression levels. This is because genes that are highly expressed tend to also be shorter, thus reducing the probability a AE site will occur. Similarly, genes that are highly expressed also tend to show strong codon bias and, as a result, the rate at which novel genes generated via gene duplication diverge from their progenitor sequence will be slower, thus reducing the probability the gene's tags will be unique.

The reason the negative association between expression level and *φ *causes the simulations to produce Φ¯
 MathType@MTEF@5@5@+=feaafiart1ev1aaatCvAUfKttLearuWrP9MDH5MBPbIqV92AaeXatLxBI9gBaebbnrfifHhDYfgasaacH8akY=wiFfYdH8Gipec8Eeeu0xXdbba9frFj0=OqFfea0dXdd9vqai=hGuQ8kuc9pgc9s8qqaq=dirpe0xb9q8qiLsFr0=vr0=vr0dc8meaabaqaciaacaGaaeqabaqabeGadaaakeaacuqHMoGrgaqeaaaa@2E3E@ values larger than the modal estimate is because the joint mode estimate of *m *for genes with no experimentally observed tags is on the zero boundary. In contrast, the simulations essentially sample from a dirichlet distribution and, consequently, will always pull a value greater than zero. Thus in the simulations genes with low expression levels and high *φ *values contribute to Φ¯
 MathType@MTEF@5@5@+=feaafiart1ev1aaatCvAUfKttLearuWrP9MDH5MBPbIqV92AaeXatLxBI9gBaebbnrfifHhDYfgasaacH8akY=wiFfYdH8Gipec8Eeeu0xXdbba9frFj0=OqFfea0dXdd9vqai=hGuQ8kuc9pgc9s8qqaq=dirpe0xb9q8qiLsFr0=vr0=vr0dc8meaabaqaciaacaGaaeqabaqabeGadaaakeaacuqHMoGrgaqeaaaa@2E3E@ more than they do in the calculation of Φ¯
 MathType@MTEF@5@5@+=feaafiart1ev1aaatCvAUfKttLearuWrP9MDH5MBPbIqV92AaeXatLxBI9gBaebbnrfifHhDYfgasaacH8akY=wiFfYdH8Gipec8Eeeu0xXdbba9frFj0=OqFfea0dXdd9vqai=hGuQ8kuc9pgc9s8qqaq=dirpe0xb9q8qiLsFr0=vr0=vr0dc8meaabaqaciaacaGaaeqabaqabeGadaaakeaacuqHMoGrgaqeaaaa@2E3E@ based on the joint mode. We have verified this idea by randomly reassigning *φ *values to each gene, thus removing any relationship between *φ *and *m*. In these simulations the posterior mode overlaps with and simulation based 95%PI for Φ¯
 MathType@MTEF@5@5@+=feaafiart1ev1aaatCvAUfKttLearuWrP9MDH5MBPbIqV92AaeXatLxBI9gBaebbnrfifHhDYfgasaacH8akY=wiFfYdH8Gipec8Eeeu0xXdbba9frFj0=OqFfea0dXdd9vqai=hGuQ8kuc9pgc9s8qqaq=dirpe0xb9q8qiLsFr0=vr0=vr0dc8meaabaqaciaacaGaaeqabaqabeGadaaakeaacuqHMoGrgaqeaaaa@2E3E@.

#### Comparison of mRNA and tag frequencies

**Figure 6:**
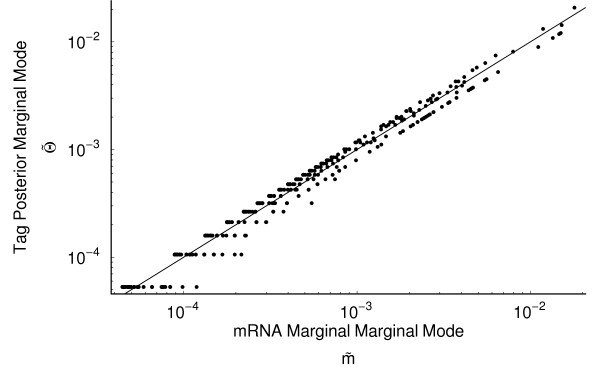
Comparison of the tag and mRNA frequency marginal modes (θ˜
 MathType@MTEF@5@5@+=feaafiart1ev1aaatCvAUfKttLearuWrP9MDH5MBPbIqV92AaeXatLxBI9gBaebbnrfifHhDYfgasaacH8akY=wiFfYdH8Gipec8Eeeu0xXdbba9frFj0=OqFfea0dXdd9vqai=hGuQ8kuc9pgc9s8qqaq=dirpe0xb9q8qiLsFr0=vr0=vr0dc8meaabaqaciaacaGaaeqabaqabeGadaaakeaaiiGacuWF4oqCgaacaaaa@2E78@ and m˜
 MathType@MTEF@5@5@+=feaafiart1ev1aaatCvAUfKttLearuWrP9MDH5MBPbIqV92AaeXatLxBI9gBaebbnrfifHhDYfgasaacH8akY=wiFfYdH8Gipec8Eeeu0xXdbba9frFj0=OqFfea0dXdd9vqai=hGuQ8kuc9pgc9s8qqaq=dirpe0xb9q8qiLsFr0=vr0=vr0dc8meaabaqaciaacaGaaeqabaqabeGadaaakeaacuWGTbqBgaacaaaa@2E1E@ respectively) during log growth phase. Data is presented on a log-log scale with a 1:1 line for reference. Genes whose tag formation cutting probability *φ*_*i *_is greater that the mean tag formation probability Φ¯
 MathType@MTEF@5@5@+=feaafiart1ev1aaatCvAUfKttLearuWrP9MDH5MBPbIqV92AaeXatLxBI9gBaebbnrfifHhDYfgasaacH8akY=wiFfYdH8Gipec8Eeeu0xXdbba9frFj0=OqFfea0dXdd9vqai=hGuQ8kuc9pgc9s8qqaq=dirpe0xb9q8qiLsFr0=vr0=vr0dc8meaabaqaciaacaGaaeqabaqabeGadaaakeaacuqHMoGrgaqeaaaa@2E3E@ are over represented in the tag pool and, consequently, occur below the 1:1 line. Conversely, genes whose tag formation cutting probability *φ*_*i *_is less that Φ¯
 MathType@MTEF@5@5@+=feaafiart1ev1aaatCvAUfKttLearuWrP9MDH5MBPbIqV92AaeXatLxBI9gBaebbnrfifHhDYfgasaacH8akY=wiFfYdH8Gipec8Eeeu0xXdbba9frFj0=OqFfea0dXdd9vqai=hGuQ8kuc9pgc9s8qqaq=dirpe0xb9q8qiLsFr0=vr0=vr0dc8meaabaqaciaacaGaaeqabaqabeGadaaakeaacuqHMoGrgaqeaaaa@2E3E@ are under represented in the tag pool and occur above the 1:1 line.

We also calculated the marginal modes for the tag frequencies, which is equivalent to standard estimates. The relative differences between the tag and mRNA pool frequencies as a function of *φ *are illustrated in Figure 3. The direct comparison between the marginal mode of tag and mRNA frequencies, θ˜i
 MathType@MTEF@5@5@+=feaafiart1ev1aaatCvAUfKttLearuWrP9MDH5MBPbIqV92AaeXatLxBI9gBaebbnrfifHhDYfgasaacH8akY=wiFfYdH8Gipec8Eeeu0xXdbba9frFj0=OqFfea0dXdd9vqai=hGuQ8kuc9pgc9s8qqaq=dirpe0xb9q8qiLsFr0=vr0=vr0dc8meaabaqaciaacaGaaeqabaqabeGadaaakeaaiiGacuWF4oqCgaacamaaBaaaleaacqWGPbqAaeqaaaaa@2FFF@ and m˜i
 MathType@MTEF@5@5@+=feaafiart1ev1aaatCvAUfKttLearuWrP9MDH5MBPbIqV92AaeXatLxBI9gBaebbnrfifHhDYfgasaacH8akY=wiFfYdH8Gipec8Eeeu0xXdbba9frFj0=OqFfea0dXdd9vqai=hGuQ8kuc9pgc9s8qqaq=dirpe0xb9q8qiLsFr0=vr0=vr0dc8meaabaqaciaacaGaaeqabaqabeGadaaakeaacuWGTbqBgaacamaaBaaaleaacqWGPbqAaeqaaaaa@2FA5@ are illustrated Figure 6. As a result these genes occur below the 1:1 line in Figure 6, illustrating how ignoring the tag formation process will lead to underestimates of these genes mRNA frequencies. In contrast, genes with higher than average tag formation probabilities, i.e. *φ*_*i *_> Φ¯^
 MathType@MTEF@5@5@+=feaafiart1ev1aaatCvAUfKttLearuWrP9MDH5MBPbIqV92AaeXatLxBI9gBaebbnrfifHhDYfgasaacH8akY=wiFfYdH8Gipec8Eeeu0xXdbba9frFj0=OqFfea0dXdd9vqai=hGuQ8kuc9pgc9s8qqaq=dirpe0xb9q8qiLsFr0=vr0=vr0dc8meaabaqaciaacaGaaeqabaqabeGadaaakeaacuqHMoGrgaqegaqcaaaa@2E4D@, are over represented in the tag pool. As a result these genes occur above the 1:1 line in Figure 6, indicating how standard methods overestimate these genes mRNA frequencies.

## Discussion

Previous approaches to analyzing SAGE data equated the sampling of the tag pool with sampling the mRNA pool (from which the tag pool was derived). In this study we developed a novel, probabilistic approach to evaluate gene expression levels of SAGE data. Our model takes into account the previously ignored tag formation process so that observations of gene tags are properly weighted by their probability of formation. Previous research has not combined all of these factors in the analysis of SAGE data resulting in significant biases in estimates of expression levels.

Our results indicate that the probability of a gene forming a SAGE tag varies greatly from gene to gene and between experiments. We find that genes with higher than average probabilities of forming SAGE tags will be over-represented in the tag pool. As a result, the mRNA abundances of these genes are over-estimated using the standard approach. Conversely, we also find that genes with lower than average probabilities of forming SAGE tags will be under-represented in the tag pool. Predictably, the mRNA abundances of these genes are under-estimated using the standard approach. The picture, however, becomes even more complex when one considers the fact that genes with low expression levels tend to have higher than average tag formation probabilities. Thus, we argue that taking inter-genic variation in tag formation probabilities into account is a required step in order to properly interpret SAGE data.

Sometimes the goal of a set of SAGE experiments is to make inferences about relative changes in mRNA expression levels between two different treatments. Even under these circumstances, accounting for the effect of tag formation will improve the quality of inferences made based on their observed frequencies. This is because the cutting efficiency *p *varies between experiments, thereby, causing the tag formation probability *φ*_*i *_and Φ¯
 MathType@MTEF@5@5@+=feaafiart1ev1aaatCvAUfKttLearuWrP9MDH5MBPbIqV92AaeXatLxBI9gBaebbnrfifHhDYfgasaacH8akY=wiFfYdH8Gipec8Eeeu0xXdbba9frFj0=OqFfea0dXdd9vqai=hGuQ8kuc9pgc9s8qqaq=dirpe0xb9q8qiLsFr0=vr0=vr0dc8meaabaqaciaacaGaaeqabaqabeGadaaakeaacuqHMoGrgaqeaaaa@2E3E@ to vary between experiments which, in turn, affect the ratio of the mRNA estimates (c.f. (11)). If SAGE data is being used an exploratory tool or to verify that a hypothesized gene is actually expressed as opposed to being a pseudo-gene, the methods developed here also have some application. Intuitively, experimentalists already know that inferences for genes lacking unique tag sites cannot be made. However, instead of classifying genes as either detectable or non-detectable through SAGE (i.e. *φ *> 0 vs *φ *= 0), our methods allow researchers to develop a more nuanced understanding of a hypothesized transcript's ability to be detected.

We find that the probability of tag formation from an mRNA depends on (a) the AE cleavage efficiency *p*, (b) the number of anchoring enzyme sites within a gene's mRNA transcript and (c) whether such tags can be unambiguously assigned to a single gene. The AE cleavage efficiency effects the distribution of tags formed from an individual gene. It might seem that, experimentally, obtaining 100% AE cleavage efficiency would be a desirable goal. However, as discussed, extremely high efficiency has drawbacks. When cleavage efficiency is 100%, only the most 3' tags or final tag will be cleaved for each transcript, resulting in a single type of tag for each gene [9]. Under such conditions, the distribution of tags formed is weighted fully with the final tag and any gene whose final tag is ambiguous will be rendered unobservable.

In contrast, as AE cleavage efficiency decreases, the distribution of tags formed is more evenly distributed, resulting in the formation of multiple tags from the set of mRNA transcripts of a single gene [9]. Thus, it is arguable that if the mRNA pool is sufficiently large, a very low AE would actually be desirable since it would likely make all genes with multiple AE sites observable and reduce the sensitivity of the analysis to errors in determining the end of the 3' UTR (see below). Experimentally, AE cleavage effciencies are significantly less than 100% and vary between experiments. This quantitative conclusion is also consistent with empirical observations that partial digestion often occurs during SAGE experiments [18].

While the AE cleavage efficiency varies between experiments, in the absence of any alternative splicing, the number and type (ambiguous vs. informative) of tags that can formed from a gene's mRNA does not. However, tag site number and type do vary from gene to gene which leads to inter-genic differences in tag formation probabilities. Because *φ*_*i *_is the sum of tagging probabilities at informative sites, removal of a AE site close to the 3' end can greatly reduce the value of *φ*_*i*. _Because many of the most 3' tags are likely to reside in the 3' UTR region, a region which is generally poorly understood and delimited, unambiguously assigning such tags to specific genes becomes increasingly problematic. Because the incorrect inclusion (exclusion) of a 3' tag erroneously elevates (depresses) the tag formation probability as an increasing function of *p*, a low AE cutting efficiency might actually be desirable in these situations. Note, however, that as AE cutting effciencies decrease, the importance of correctly determining the 5' UTR boundary increases.

In general, the more AE sites a gene contains, the larger is its value of *φ*_*i*_. Because shorter genes tend to have fewer AE sites, there is a positive relationship between gene length and *φ*_*i *_(data now shown). Interestingly, in *Saccharomyces cerevisiae *in either L and S phases, *m*_*i *_and *φ*_*i *_have a loose negative correlation with one another (*r *= -0.041 and -0.035, *t *= -3.21 and -2.74 and *p *< 0.0001, and *p *< 0.005, respectively). This indicates that highly expressed genes tend to be shorter in length and therefore have fewer potential AE sites. Hence, in general, standard estimates, θ^i
 MathType@MTEF@5@5@+=feaafiart1ev1aaatCvAUfKttLearuWrP9MDH5MBPbIqV92AaeXatLxBI9gBaebbnrfifHhDYfgasaacH8akY=wiFfYdH8Gipec8Eeeu0xXdbba9frFj0=OqFfea0dXdd9vqai=hGuQ8kuc9pgc9s8qqaq=dirpe0xb9q8qiLsFr0=vr0=vr0dc8meaabaqaciaacaGaaeqabaqabeGadaaakeaaiiGacuWF4oqCgaqcamaaBaaaleaacqWGPbqAaeqaaaaa@3000@, will under-estimate the expression levels for highly expressed genes. For example, the tag count for gene YKL152C is 228 in the L-phase. There is a single AE site for this gene. Therefore, its tagging probability (*φ*_*i*_) is 0.56 in the L phase, which means a correction of ~ 15% in its expression level (m^
 MathType@MTEF@5@5@+=feaafiart1ev1aaatCvAUfKttLearuWrP9MDH5MBPbIqV92AaeXatLxBI9gBaebbnrfifHhDYfgasaacH8akY=wiFfYdH8Gipec8Eeeu0xXdbba9frFj0=OqFfea0dXdd9vqai=hGuQ8kuc9pgc9s8qqaq=dirpe0xb9q8qiLsFr0=vr0=vr0dc8meaabaqaciaacaGaaeqabaqabeGadaaakeaacuWGTbqBgaqcaaaa@2E1F@ = 0.022 versus θ^
 MathType@MTEF@5@5@+=feaafiart1ev1aaatCvAUfKttLearuWrP9MDH5MBPbIqV92AaeXatLxBI9gBaebbnrfifHhDYfgasaacH8akY=wiFfYdH8Gipec8Eeeu0xXdbba9frFj0=OqFfea0dXdd9vqai=hGuQ8kuc9pgc9s8qqaq=dirpe0xb9q8qiLsFr0=vr0=vr0dc8meaabaqaciaacaGaaeqabaqabeGadaaakeaaiiGacuWF4oqCgaqcaaaa@2E79@ = 0.019). Thus accounting for variation in tag formation probabilities *φ *becomes especially important when trying to measure the saturation of microarray data by comparing it to SAGE data (e.g. [17]).

To incorporate variation in tag formation probabilities we took a decidedly Bayesian approach, modeling the sampling of tag frequencies as a multinomial process and using a Dirichlet prior for mRNA abundances. Despite the complexity of the tag formation process, we were able obtain a number of analytic results or approximations which were verified using simulation. More importantly, our analytic results offer a useful contrast of the assumptions involved in data analysis based on a Bayesian and Frequentist approaches.

One main drawback with the Bayesian approach is the large amount of information that is assumed already known when using a flat, uninformative prior. With a Dirichlet prior, the number of 'prior observations' implicit in a flat prior is equal to the number of genes observable with SAGE [20]. For the *Saccharomyces cerevisiae *databases used here, this number is on the order of five thousand. This number of observations is only a few fold below the number of observed informative tags in a given SAGE dataset. Alternatively, one could use other, less weighty priors such as *α*_*i *_= 1/*n*. Doing so, however, results in the large, inconsistent shifts in the mode of the marginal posterior distribution with tag numbers when the observed number of tags are small (i.e. ≤ 3). This prior also produces singularities in certain ranges of Φ¯
 MathType@MTEF@5@5@+=feaafiart1ev1aaatCvAUfKttLearuWrP9MDH5MBPbIqV92AaeXatLxBI9gBaebbnrfifHhDYfgasaacH8akY=wiFfYdH8Gipec8Eeeu0xXdbba9frFj0=OqFfea0dXdd9vqai=hGuQ8kuc9pgc9s8qqaq=dirpe0xb9q8qiLsFr0=vr0=vr0dc8meaabaqaciaacaGaaeqabaqabeGadaaakeaacuqHMoGrgaqeaaaa@2E3E@, leading additional numerical complications when estimating its value.

In contrast, examination of the posterior marginal distributions illustrates how a Bayesian framework differs from the Frequentist approach. More specifically, the Frequentist approach, which focuses on the marginal likelihood of a single gene is analogous to the Bayesian posterior marginal distribution with the prior parameters of *α *= *β *= 1. In the Bayesian framework such a prior is undesirable because it results in an inconsistency. Specifically, in a Bayesian framework *α *= *α*_*i *_and β=∑j≠iαj
 MathType@MTEF@5@5@+=feaafiart1ev1aaatCvAUfKttLearuWrP9MDH5MBPbIqV92AaeXatLxBI9gBaebbnrfifHhDYfgasaacH8akY=wiFfYdH8Gipec8Eeeu0xXdbba9frFj0=OqFfea0dXdd9vqai=hGuQ8kuc9pgc9s8qqaq=dirpe0xb9q8qiLsFr0=vr0=vr0dc8meaabaqaciaacaGaaeqabaqabeGadaaakeaaiiGacqWFYoGycqGH9aqpdaaeqaqaaiab=f7aHnaaBaaaleaacqWGQbGAaeqaaaqaaiabdQgaQjabgcMi5kabdMgaPbqab0GaeyyeIuoaaaa@38D4@, where *i *is the focal gene. So to have *α *= *α*_*i *_= 1 and *β *= 1 for one gene implies that *α*_*j *_cannot equal 1 for any of the other genes, yet that is exactly what is assumed when analyzing these other genes.

Conceivably, if we can estimate the prior distribution empirically, we may further improve estimation of expression levels and avoid some of the problems encountered with regard to the large amount of 'prior observations' implicit in our flat, uninformative prior. However, the implementation of such an approach would be difficult since it would entail integration and possibly maximization over the the very high-dimensional Dirichlet prior distribution.

In comparison to other Bayesian methods developed to analyze SAGE data, Thygesen and Zwinderman [10] use a combination of Bayesian and maximum likelihood approaches to model the distribution of tags arising from SAGE analysis. Instead of modeling observed mRNA proportions, they used a hierarchical Poisson model with a gamma prior to model the observed mRNA counts. The main thrust of the paper is to fit the hierarchical Poisson model using maximum likelihood although some discussion of Bayesian inference is also included. The paper also seems to view the counts as independent and identically distributed observations making the additional variation of the hierarchical Poisson model useful.

The analysis of Morris, Baggerly and Coombes [11] is closest in spirit to our work. They directly apply a Bayesian multinomial-Dirichlet model to the observed vector of tag counts. This approach improves upon most earlier work by considering simultaneous inference on all proportions *m*_*i*_. They provide a simple computationally tractable approach and consider the result of the statistical shrinkage effect which offers improved estimates for proportions with low tag counts while underestimating the expression proportions for tags with large counts. This leads them to propose a mixture Dirichlet prior in order to mitigate the propensity to underestimate highly expressed genes. However, they do not consider the variation in tag formation probabilities which is the main focus of this paper.

## Conclusion

Previous studies of SAGE data have implicitly assumed that the tag pool is an unbiased representation of the mRNA pool. By building a mechanistic model of tag formation we show how this assumption only holds when all genes have the same tag formation probability and, more importantly, how to properly adjust one's inferences according to the tag formation probability of the gene relative to the entire mRNA population. We believe that this work is a valuable addition to the existing methods for SAGE data analysis and, given its probabilistic nature, can be integrated into other studies of SAGE data.

## Methods

### Sources of data

Yeast transcripts were parsed out from chromosomal sequences downloaded from the Saccharomyces Genome Database on July 13, 2006 [21]. SAGE data were also obtained from the Saccharomyces Genome Database. Tags which could not be mapped to the transcripts of any known gene or, conversely, could be mapped to the transcripts of multiple genes were excluded from our analysis.

### Data processing implementations

All computations were implemented using Linux Fedora 4 and 5. All code (e.g. PERL scripts, R routines, and Mathematica routines) are released under GPL V2 and without warranty. This code is available in Additional File 10 or at .

#### Processing of sequence data

PERL scripts were used for identification of potential AE sites and parsing of transcripts.

#### Numerical calculations

Numerical calculations to solve for Φ¯
 MathType@MTEF@5@5@+=feaafiart1ev1aaatCvAUfKttLearuWrP9MDH5MBPbIqV92AaeXatLxBI9gBaebbnrfifHhDYfgasaacH8akY=wiFfYdH8Gipec8Eeeu0xXdbba9frFj0=OqFfea0dXdd9vqai=hGuQ8kuc9pgc9s8qqaq=dirpe0xb9q8qiLsFr0=vr0=vr0dc8meaabaqaciaacaGaaeqabaqabeGadaaakeaacuqHMoGrgaqeaaaa@2E3E@ and the posterior marginal distributions for mRNA frequencies were done using Mathematica [22].

#### Simulation

Our simulation utilized a Gibbs Sampling approach. Background on this technique can be found in [20]. The model proposes that an initial population of cDNA *G *= (*g*_*1*_, ..., *g*_*n*_) is generated based upon a multinomial population of size *N *with proportions *m*_*i*_.

P(G|α)~(Ng1,g2,...,gk)m1g1m2g2,...,mkgn
 MathType@MTEF@5@5@+=feaafiart1ev1aaatCvAUfKttLearuWrP9MDH5MBPbIqV92AaeXatLxBI9gBaebbnrfifHhDYfgasaacH8akY=wiFfYdH8Gipec8Eeeu0xXdbba9frFj0=OqFfea0dXdd9vqai=hGuQ8kuc9pgc9s8qqaq=dirpe0xb9q8qiLsFr0=vr0=vr0dc8meaabaqaciaacaGaaeqabaqabeGadaaakeaacqWGqbaucqGGOaakcqWGhbWrcqGG8baFiiGacqWFXoqycqGGPaqkcqGG+bGFdaqadaqaauaabeqaceaaaeaacqWGobGtaeaacqWGNbWzdaWgaaWcbaGaeGymaedabeaakiabcYcaSiabdEgaNnaaBaaaleaacqaIYaGmaeqaaOGaeiilaWIaeiOla4IaeiOla4IaeiOla4IaeiilaWIaem4zaC2aaSbaaSqaaiabdUgaRbqabaaaaaGccaGLOaGaayzkaaGaemyBa02aa0baaSqaaiabigdaXaqaaiabdEgaNnaaBaaameaacqaIXaqmaeqaaaaakiabd2gaTnaaDaaaleaacqaIYaGmaeaacqWGNbWzdaWgaaadbaGaeGOmaidabeaaaaGccqGGSaalcqGGUaGlcqGGUaGlcqGGUaGlcqGGSaalcqWGTbqBdaqhaaWcbaGaem4AaSgabaGaem4zaC2aaSbaaWqaaiabd6gaUbqabaaaaaaa@597B@

Uncertainty about the population size *N *is addressed by assuming that it follows a Poisson distribution. Uncertainty in the proportions *m*_*i *_are assumed to follow a Dirichlet distribution, a generalization of the Beta. Finally the number of tagged counts observed for gene *i *follows a binomial distribution, *T*_*i *_~ Bin (*g*_*i*_, *φ*_*i*_). Details on conditional distributions and a discussion of implementation of the Gibbs Sampler can be found in [23].

### Identification of transcripts boundaries

We parsed out 2370 genes with annotated UTRs from the segment table generated from poly-A RNA by tiling arrays [19]. Among them, 28 genes show inconsistent nomenclature when compared with the version of SGD data set that we used. For simplicity, we ignored these 28 genes. Hence, we obtained UTR coordinates for 2342 genes. The median 5' and 3' UTR lengths are 70 bp and 94 bp respectively. We rounded the numbers and parsed out 70 bp as upstream and 95 bp downstream for the remaining genes.

Because the 3' boundaries of transcripts are more important to SAGE data analysis than the 5' boundaries, we tried 70 bp 5' UTR and 250 bp 3' UTR based on a different experimental data set [24].

## Abbreviations

SAGE Serial analysis of gene expression

AE Anchoring Enzyme

UTR Untranslated Regions

PI Probability Interval

bp base pairs

## Authors' contributions

MAG initiated the project, derived the distributions with RZ, derived the approximations, and wrote the later drafts. HQ wrote the initial drafts, implemented the PERL scripts, and verified the approximations. RZ implemented the simulations, helped derive the distributions, and also verified the approximations. All authors have read and approved this final manuscript.

## Additional files

Supplementary files present the results for the 6179 genes in *Saccharomyces cerevisiae *with unique tags. All calculations are under the assumption of a flat, uninformative prior of *α*_*i *_= 1 for all genes. SAGE data comes from three experiments (L, S, and G2M) published in [9]. Updated versions of all tables are available at .

## Appendices

### A. Estimation of the global cleavage efficiency of the anchoring enzyme

We designed a likelihood approach to estimate *p*, the global cleavage efficiency of AE. Remarkably, *p *can be estimated when only considering the coding regions of the transcripts, as we will show below. The coding regions are much better annotated than 5' and 3' UTRs. In fact, we do not know the UTR boundaries for most of the yeast transcripts. Hence, the estimation of *p *based on coding regions should be more accurate than the estimation based on transcripts.

As seen in Figure 1, we can assume that the *j*^th ^potential AE site is the last site within the coding region, without loss of generality. The conditional tagging probability at the *j*^th ^site given only observations in the coding region is

P(cut at site j|no cuts at sites 3' to site j)=p(1−p)(k−j)(1−p)(k−j)=p,
 MathType@MTEF@5@5@+=feaafiart1ev1aaatCvAUfKttLearuWrP9MDH5MBPbIqV92AaeXatLxBI9gBaebbnrfifHhDYfgasaacH8akY=wiFfYdH8Gipec8Eeeu0xXdbba9frFj0=OqFfea0dXdd9vqai=hGuQ8kuc9pgc9s8qqaq=dirpe0xb9q8qiLsFr0=vr0=vr0dc8meaabaqaciaacaGaaeqabaqabeGadaaakeaacqWGqbaucqGGOaakcqqGJbWycqqG1bqDcqqG0baDcqqGGaaicqqGHbqycqqG0baDcqqGGaaicqqGZbWCcqqGPbqAcqqG0baDcqqGLbqzcqqGGaaicqWGQbGAcqGG8baFcqqGUbGBcqqGVbWBcqqGGaaicqqGJbWycqqG1bqDcqqG0baDcqqGZbWCcqqGGaaicqqGHbqycqqG0baDcqqGGaaicqqGZbWCcqqGPbqAcqqG0baDcqqGLbqzcqqGZbWCcqqGGaaicqaIZaWmcqGGNaWjcqqGGaaicqqG0baDcqqGVbWBcqqGGaaicqqGZbWCcqqGPbqAcqqG0baDcqqGLbqzcqqGGaaicqWGQbGAcqGGPaqkcqGH9aqpdaWcaaqaaiabdchaWjabcIcaOiabigdaXiabgkHiTiabdchaWjabcMcaPmaaCaaaleqabaGaeiikaGIaem4AaSMaeyOeI0IaemOAaOMaeiykaKcaaaGcbaGaeiikaGIaeGymaeJaeyOeI0IaemiCaaNaeiykaKYaaWbaaSqabeaacqGGOaakcqWGRbWAcqGHsislcqWGQbGAcqGGPaqkaaaaaOGaeyypa0JaemiCaaNaeiilaWcaaa@7F05@

which is the same value for tagging probability at the last site, the *k*^th ^site, for the full transcript. It can be seen that the conditional tagging probability at the (*j *- 1)^th ^site is *p*(1 - *p*), which equals the tagging probability at the (*k *- 1)^th ^site for the full-length transcript. Similar conclusions can be reached for all other potential AE sites within the coding region. Hence, the tagging probabilities at potential AE sites within coding regions given only observations at the coding regions are identical to tagging probabilities at equivalent sites for full-length transcripts.

Now, we can proceed to use a likelihood approach to estimate *p *using tags observed only from the coding regions. To avoid confusion with other analysis based on full-length transcripts, we will use slightly different notations for indexes here. We consider the total number of potential AE sites in the coding region for gene *i *is k′i
 MathType@MTEF@5@5@+=feaafiart1ev1aaatCvAUfKttLearuWrP9MDH5MBPbIqV92AaeXatLxBI9gBaebbnrfifHhDYfgasaacH8akY=wiFfYdH8Gipec8Eeeu0xXdbba9frFj0=OqFfea0dXdd9vqai=hGuQ8kuc9pgc9s8qqaq=dirpe0xb9q8qiLsFr0=vr0=vr0dc8meaabaqaciaacaGaaeqabaqabeGadaaakeaacuWGRbWAgaqbamaaBaaaleaacqWGPbqAaeqaaaaa@2F9E@.

For the *i*^th ^gene, we consider the observed tags at all the AE sites, T→i={Ti,1,Ti,2,...,Ti,j,...,Ti,k′i}
 MathType@MTEF@5@5@+=feaafiart1ev1aaatCvAUfKttLearuWrP9MDH5MBPbIqV92AaeXatLxBI9gBaebbnrfifHhDYfgasaacH8akY=wiFfYdH8Gipec8Eeeu0xXdbba9frFj0=OqFfea0dXdd9vqai=hGuQ8kuc9pgc9s8qqaq=dirpe0xb9q8qiLsFr0=vr0=vr0dc8meaabaqaciaacaGaaeqabaqabeGadaaakeaacuWGubavgaWcamaaBaaaleaacqWGPbqAaeqaaOGaeyypa0Jaei4EaSNaemivaq1aaSbaaSqaaiabdMgaPjabcYcaSiabigdaXaqabaGccqGGSaalcqWGubavdaWgaaWcbaGaemyAaKMaeiilaWIaeGOmaidabeaakiabcYcaSiabc6caUiabc6caUiabc6caUiabcYcaSiabdsfaunaaBaaaleaacqWGPbqAcqGGSaalcqWGQbGAaeqaaOGaeiilaWIaeiOla4IaeiOla4IaeiOla4IaeiilaWIaemivaq1aaSbaaSqaaiabdMgaPjabcYcaSiqbdUgaRzaafaWaaSbaaWqaaiabdMgaPbqabaaaleqaaOGaeiyFa0haaa@5203@ are drawn from a total of Ti,total=∑j=1k′iTi,j
 MathType@MTEF@5@5@+=feaafiart1ev1aaatCvAUfKttLearuWrP9MDH5MBPbIqV92AaeXatLxBI9gBaebbnrfifHhDYfgasaacH8akY=wiFfYdH8Gipec8Eeeu0xXdbba9frFj0=OqFfea0dXdd9vqai=hGuQ8kuc9pgc9s8qqaq=dirpe0xb9q8qiLsFr0=vr0=vr0dc8meaabaqaciaacaGaaeqabaqabeGadaaakeaacqWGubavdaWgaaWcbaGaemyAaKMaeiilaWIaemiDaqNaem4Ba8MaemiDaqNaemyyaeMaemiBaWgabeaakiabg2da9maaqadabaGaemivaq1aaSbaaSqaaiabdMgaPjabcYcaSiabdQgaQbqabaaabaGaemOAaOMaeyypa0JaeGymaedabaGafm4AaSMbauaadaWgaaadbaGaemyAaKgabeaaa0GaeyyeIuoaaaa@457B@ of tags based on a multinomial distribution. The probability of a "successful" draw at the *j*^th ^site is

p(1−p)(k′i−j)(1−(1−p)k′i).
 MathType@MTEF@5@5@+=feaafiart1ev1aaatCvAUfKttLearuWrP9MDH5MBPbIqV92AaeXatLxBI9gBaebbnrfifHhDYfgasaacH8akY=wiFfYdH8Gipec8Eeeu0xXdbba9frFj0=OqFfea0dXdd9vqai=hGuQ8kuc9pgc9s8qqaq=dirpe0xb9q8qiLsFr0=vr0=vr0dc8meaabaqaciaacaGaaeqabaqabeGadaaakeaadaWcaaqaaiabdchaWjabcIcaOiabigdaXiabgkHiTiabdchaWjabcMcaPmaaCaaaleqabaGaeiikaGIafm4AaSMbauaadaWgaaadbaGaemyAaKgabeaaliabgkHiTiabdQgaQjabcMcaPaaaaOqaaiabcIcaOiabigdaXiabgkHiTiabcIcaOiabigdaXiabgkHiTiabdchaWjabcMcaPmaaCaaaleqabaGafm4AaSMbauaadaWgaaadbaGaemyAaKgabeaaaaGccqGGPaqkaaGaeiOla4caaa@46E3@

Using a flat, uninformative prior of *φ*(*P*) ~ Beta(1, 1), the posterior probability distribution of *p *given the observed tag distributions across *i *genes is

P(p|T→i)∝∏i∏j=1k′i(p(1−p)(k′i−j)1−(1−p)k′i)Ti,j,j∈(informative AE sites)
 MathType@MTEF@5@5@+=feaafiart1ev1aaatCvAUfKttLearuWrP9MDH5MBPbIqV92AaeXatLxBI9gBaebbnrfifHhDYfgasaacH8akY=wiFfYdH8Gipec8Eeeu0xXdbba9frFj0=OqFfea0dXdd9vqai=hGuQ8kuc9pgc9s8qqaq=dirpe0xb9q8qiLsFr0=vr0=vr0dc8meaabaqaciaacaGaaeqabaqabeGadaaakeaafaqabeqacaaabaGaemiuaaLaeiikaGIaemiCaaNaeiiFaWNafmivaqLbaSaadaWgaaWcbaGaemyAaKgabeaakiabcMcaPiabg2Hi1oaarafabaWaaebCaeaadaqadaqaamaalaaabaGaemiCaaNaeiikaGIaeGymaeJaeyOeI0IaemiCaaNaeiykaKYaaWbaaSqabeaacqGGOaakcuWGRbWAgaqbamaaBaaameaacqWGPbqAaeqaaSGaeyOeI0IaemOAaOMaeiykaKcaaaGcbaGaeGymaeJaeyOeI0IaeiikaGIaeGymaeJaeyOeI0IaemiCaaNaeiykaKYaaWbaaSqabeaacuWGRbWAgaqbamaaBaaameaacqWGPbqAaeqaaaaaaaaakiaawIcacaGLPaaaaSqaaiabdQgaQjabg2da9iabigdaXaqaaiqbdUgaRzaafaWaaSbaaWqaaiabdMgaPbqabaaaniabg+GivdaaleaacqWGPbqAaeqaniabg+GivdGcdaahaaWcbeqaaiabdsfaunaaBaaameaacqWGPbqAcqGGSaalcqWGQbGAaeqaaaaakiabcYcaSaqaaiabdQgaQjabgIGiolabcIcaOiabbMgaPjabb6gaUjabbAgaMjabb+gaVjabbkhaYjabb2gaTjabbggaHjabbsha0jabbMgaPjabbAha2jabbwgaLjabbccaGiabbgeabjabbweafjabbccaGiabbohaZjabbMgaPjabbsha0jabbwgaLjabbohaZjabcMcaPaaaaaa@8039@

which can be evaluated numerically.

Using eqn. (A2) we calculated the posterior distributions for *p *for *Saccharomyces cerevisiae *under three different conditions, log growth, S phase-arrested, and G2M phase-arrested using the SAGE data from [9]. As shown in Fig 2, posterior distributions of the AE cleavage efficiency *p *varies considerably between experiments. This variation should be taken into account when estimating the actual expression levels, and thus highlights the utility of our modeling approach. For each experiment, we calculated the tagging probability *φ*_*i *_using the mode estimate of *p *for that experiment. As a result, the tagging probability of a given gene varies between experiments. The estimations and their 95% PI are provided in Table 2.

### B. Solution and approximation of the marginal mode of *m*_*i*_

Taking the log of eqn. 12 and differentiating it with respect to *m*_*i *_gives,

ddmi(ln⁡(f(m|T→,φ→)))=αimi+φΦ¯T0−Ti1−φΦ¯mi.
 MathType@MTEF@5@5@+=feaafiart1ev1aaatCvAUfKttLearuWrP9MDH5MBPbIqV92AaeXatLxBI9gBaebbnrfifHhDYfgasaacH8akY=wiFfYdH8Gipec8Eeeu0xXdbba9frFj0=OqFfea0dXdd9vqai=hGuQ8kuc9pgc9s8qqaq=dirpe0xb9q8qiLsFr0=vr0=vr0dc8meaabaqaciaacaGaaeqabaqabeGadaaakeaadaWcaaqaaiabdsgaKbqaaiabdsgaKjabd2gaTnaaBaaaleaacqWGPbqAaeqaaaaakmaabmaabaGagiiBaWMaeiOBa42aaeWaaeaacqWGMbGzdaqadaqaaiabd2gaTnaaeeaabaGafmivaqLbaSaacqGGSaaliiGacuWFgpGzgaWcaaGaay5bSdaacaGLOaGaayzkaaaacaGLOaGaayzkaaaacaGLOaGaayzkaaGaeyypa0ZaaSaaaeaacqWFXoqydaWgaaWcbaGaemyAaKgabeaaaOqaaiabd2gaTnaaBaaaleaacqWGPbqAaeqaaaaakiabgUcaRmaalaaabaGae8NXdygabaGafuOPdyKbaebaaaWaaSaaaeaacqWGubavdaWgaaWcbaGaeGimaadabeaakiabgkHiTiabdsfaunaaBaaaleaacqWGPbqAaeqaaaGcbaGaeGymaeJaeyOeI0YaaSaaaeaacqWFgpGzaeaacuqHMoGrgaqeaaaacqWGTbqBdaWgaaWcbaGaemyAaKgabeaaaaGccqGGUaGlaaa@5C80@

Note that we are ignoring the dependence of Φ¯
 MathType@MTEF@5@5@+=feaafiart1ev1aaatCvAUfKttLearuWrP9MDH5MBPbIqV92AaeXatLxBI9gBaebbnrfifHhDYfgasaacH8akY=wiFfYdH8Gipec8Eeeu0xXdbba9frFj0=OqFfea0dXdd9vqai=hGuQ8kuc9pgc9s8qqaq=dirpe0xb9q8qiLsFr0=vr0=vr0dc8meaabaqaciaacaGaaeqabaqabeGadaaakeaacuqHMoGrgaqeaaaa@2E3E@ on *m*_*i*_. Setting eqn. (B1) to zero and solving for *m*_*i *_yields,

m^i=x(1−(1−y))2
 MathType@MTEF@5@5@+=feaafiart1ev1aaatCvAUfKttLearuWrP9MDH5MBPbIqV92AaeXatLxBI9gBaebbnrfifHhDYfgasaacH8akY=wiFfYdH8Gipec8Eeeu0xXdbba9frFj0=OqFfea0dXdd9vqai=hGuQ8kuc9pgc9s8qqaq=dirpe0xb9q8qiLsFr0=vr0=vr0dc8meaabaqaciaacaGaaeqabaqabeGadaaakeaacuWGTbqBgaqcamaaBaaaleaacqWGPbqAaeqaaOGaeyypa0ZaaSaaaeaacqWG4baEcqGGOaakcqaIXaqmcqGHsisldaGcaaqaaiabcIcaOiabigdaXiabgkHiTiabdMha5jabcMcaPaWcbeaakiabcMcaPaqaaiabikdaYaaaaaa@3BEF@

where

x=φ(T0+α−1)+Φ(Ti+α−1+β−1)y=4(Ti+α−1)(T0+α−1+β−1)x2.
 MathType@MTEF@5@5@+=feaafiart1ev1aaatCvAUfKttLearuWrP9MDH5MBPbIqV92AaeXatLxBI9gBaebbnrfifHhDYfgasaacH8akY=wiFfYdH8Gipec8Eeeu0xXdbba9frFj0=OqFfea0dXdd9vqai=hGuQ8kuc9pgc9s8qqaq=dirpe0xb9q8qiLsFr0=vr0=vr0dc8meaabaqaciaacaGaaeqabaqabeGadaaakeaafaqaaeGabaaabaGaemiEaGNaeyypa0dcciGae8NXdyMaeiikaGIaemivaq1aaSbaaSqaaiabicdaWaqabaGccqGHRaWkcqWFXoqycqGHsislcqaIXaqmcqGGPaqkcqGHRaWkcqqHMoGrcqGGOaakcqWGubavdaWgaaWcbaGaemyAaKgabeaakiabgUcaRiab=f7aHjabgkHiTiabigdaXiabgUcaRiab=j7aIjabgkHiTiabigdaXiabcMcaPaqaaiabdMha5jabg2da9maalaaabaGaeGinaqJaeiikaGIaemivaq1aaSbaaSqaaiabdMgaPbqabaGccqGHRaWkcqWFXoqycqGHsislcqaIXaqmcqGGPaqkcqGGOaakcqWGubavdaWgaaWcbaGaeGimaadabeaakiabgUcaRiab=f7aHjabgkHiTiabigdaXiabgUcaRiab=j7aIjabgkHiTiabigdaXiabcMcaPaqaaiabdIha4naaCaaaleqabaGaeGOmaidaaaaakiabc6caUaaaaaa@6570@

Taking a first order Taylor series approximation of this solution around *y *= 0 gives,

mi=y4(T0+α−1+β−1)+o[y2]≈Ti+αi−1T0φiΦ¯+α0−n.
 MathType@MTEF@5@5@+=feaafiart1ev1aaatCvAUfKttLearuWrP9MDH5MBPbIqV92AaeXatLxBI9gBaebbnrfifHhDYfgasaacH8akY=wiFfYdH8Gipec8Eeeu0xXdbba9frFj0=OqFfea0dXdd9vqai=hGuQ8kuc9pgc9s8qqaq=dirpe0xb9q8qiLsFr0=vr0=vr0dc8meaabaqaciaacaGaaeqabaqabeGadaaakeaafaqadeGabaaabaGaemyBa02aaSbaaSqaaiabdMgaPbqabaGccqGH9aqpdaWcaaqaaiabdMha5bqaaiabisda0iabcIcaOiabdsfaunaaBaaaleaacqaIWaamaeqaaOGaey4kaSccciGae8xSdeMaeyOeI0IaeGymaeJaey4kaSIae8NSdiMaeyOeI0IaeGymaeJaeiykaKcaaiabgUcaRiabd+gaVjabcUfaBjabdMha5naaCaaaleqabaGaeGOmaidaaOGaeiyxa0fabaGaeyisIS7aaSaaaeaacqWGubavdaWgaaWcbaGaemyAaKgabeaakiabgUcaRiab=f7aHnaaBaaaleaacqWGPbqAaeqaaOGaeyOeI0IaeGymaedabaGaemivaq1aaSbaaSqaaiabicdaWaqabaGcdaWcaaqaaiab=z8aMnaaBaaaleaacqWGPbqAaeqaaaGcbaGafuOPdyKbaebaaaGaey4kaSIae8xSde2aaSbaaSqaaiabicdaWaqabaGccqGHsislcqWGUbGBaaGaeiOla4caaaaa@5FE9@

This value is positive so long as *T*_*i *_+ *α*_*i *_- 1 > 0. Otherwise, the mode is at the boundary *m *= 0.

## Supplementary Material

Additional file 1 Marginal posterior percentiles for S experiments. Table of percentile values for the marginal posterior distribution of mRNA frequencies of *S. cerevisiae *during stationary phase. Click here for file

Additional file 2 Marginal posterior percentiles for L experiments. Table of percentile values for the marginal posterior distribution of mRNA frequencies of *S. cerevisiae *during log growth phase. Click here for file

Additional file 3 Marginal posterior percentiles for G2M experiments. Table of percentile values for the marginal posterior distribution of mRNA frequencies of *S. cerevisiae *during G2M phase. Click here for file

Additional file 4 Summary statistics of posterior distributions for S experiments. Table of posterior joint modes and marginal modes, median, variance and 95%PI of mRNA frequencies of *S. cerevisiae *during stationary phase. Click here for file

Additional file 5 Summary statistics of posterior distributions for L experiments. Table of posterior joint modes and marginal modes, median, variance and 95%PI of mRNA frequencies of *S. cerevisiae *during log growth phase. Click here for file

Additional file 6 Summary statistics of posterior distributions for G2M experiments. Table of posterior joint modes and marginal modes, median, variance and 95%PI of mRNA frequencies of *S. cerevisiae *during G2M phase. Click here for file

Additional file 7 Tag information for S experiment. Table of total AE sites, unique AE sites, tag counts, *φ *and joint mode values for stationary phase experimental data in [9]. Click here for file

Additional file 8 Tag information for L experiment. Table of total AE sites, unique AE sites, tag counts, *φ *and joint mode values for log growth experimental data in [9]. Click here for file

Additional file 9 Tag information for G2M experiment. Table of total AE sites, unique AE sites, tag counts, *φ *and joint mode values for G2M growth experimental data in [9]. Click here for file

Additional file 10 Computer code. Tar archive of PERL scripts, R routines, and Mathematica routines used in this work. All code is released under GPL V2 and without warranty. Click here for file

## References

[B1] Velculescu VE, Zhang L, Vogelstein B, Kinzler KW (1995). Serial Analysis of Gene Expression. Science.

[B2] Kuznetsov VA, Knott GD, Bonner RF (2002). General Statistics of Stochastic Process of Gene Expression in Eukaryotic Cells. Genetics.

[B3] Zhang L, Zhou W, Velculescu VE, Kern SE, Hruban RH, Hamilton SR, Vogelstein B, Kinzler KW (1997). Gene expression profiles in normal and cancer cells. Science.

[B4] Vencio RZN, Brentani H, Pereira CAB (2003). Using credibility intervals instead of hypothesis tests in SAGE analysis. Bioinformatics.

[B5] Madden SL, Galella EA, Zhu J, Bertelsen AH, Beaudry GA (1997). SAGE transcript profiles for p53-dependent growth regulation. Oncogene.

[B6] Audic S, Claverie JM (1997). The significance of digital gene expression profiles. Genome Res.

[B7] Stern MD, Anisimov SV, Boheler KR (2003). Can transcriptome size be estimated from SAGE catalogs?. Bioinformatics.

[B8] Cai L, Huang H, Blackshaw S, Liu J, Cepko C, Wong W (2004). Clustering analysis of SAGE data using a Poisson approach. Genome Biology.

[B9] Velculescu VE, Zhang L, Zhou W, Vogelstein J, Basrai MA, Bassett JDE, Hieter P, Vogelstein B, Kinzler KW (1997). Characterization of the yeast transcriptome. Cell.

[B10] Thygesen HH, Zwinderman AH (2006). Modeling Sage data with a truncated gamma-Poisson model. BMC Bioinformatics.

[B11] Morris JS, Baggerly KA, Coombes KR (2003). Bayesian shrinkage estimation of the relative abundance of mRNA transcripts using SAGE. Biometrics.

[B12] Baggerly KA, Deng L, Morris JS, Aldaz CM (2003). Differential expression in SAGE: accounting for normal between-library variation. Bioinform.

[B13] Vencio RZN, Brentani H, Patrao DFC, Pereira CAB (2004). Bayesian model accounting for within-class biological variability in Serial Analysis of Gene Expression (SAGE). BMC Bioinformatics.

[B14] Colinge J, Feger G (2001). Detecting the impact of sequencing errors on SAGE data. Bioinformatics.

[B15] Akmaev VR, Wang CJ (2004). Correction of sequence-based artifacts in serial analysis of gene expression. Bioinformatics.

[B16] Beissbarth T, Hyde L, Smyth GK, Job C, Boon WM, Tan SS, Scott HS, Speed TP (2004). Statistical modeling of sequencing errors in SAGE libraries. Bioinformatics.

[B17] Beyer A, Hollunder J, Nasheuer HP, Wilhelm T (2004). Post-transcriptional Expression Regulation in the Yeast *Saccharomyces cerevisiae *on a Genomic Scale. Mol Cell Proteomics.

[B18] Harbers M, Carninci P (2005). Tag-based approaches for transcriptome research and genome annotation. Nat Methods.

[B19] David L, Huber W, Granovskaia M, Toedling J, Palm CJ, Bofkin L, Jones T, Davis RW, Steinmetz LM (2006). A high-resolution map of transcription in the yeast genome. PNAS.

[B20] Gelman A, Carlin JB, Stern HS, Rubin DB (2004). Bayesian Data Analysis Texts in Statistical Science.

[B21] Dolinski K, Balakrishnan R, Christie KR, Costanzo MC, Dwight SS, Engel SR, Fisk DG, Hirschman JE, Hong EL, Issel-Tarver L, Sethuraman A, Theesfeld CL, Binkley G, Lane C, Schroeder M, Dong S, Weng S, Andrada R, Botstein D, Cherry JM (2003). Saccharomyces Genome Database. ftp://ftp.yeastgenome.org/yeast/.

[B22] Wolfram Research Inc (2005). Mathematica.

[B23] Zaretzki R, Gilchrist MA, Briggs WM, Armagan A Improved Estimates of the Relative Abundance of mRNA using SAGE, a Gibbs Sampling Approach. Biometrics.

[B24] Hurowitz EH, Brown PO (2003). Genome-wide analysis of mRNA lengths in *Saccharomyces cerevisiae*. Genome Biol.

